# Three New Aporphine Alkaloids with Glucose Consumption Increase Activity from *Cassytha filiformis*

**DOI:** 10.3390/molecules30234544

**Published:** 2025-11-25

**Authors:** Caiyun Zhang, Yongrui Lin, Licui Xie, Yiru Wang, Zhiren Xie, Lin Dong, Yanhui Fu

**Affiliations:** 1 College of Chemistry and Chemical Engineering, Hainan Normal University, Haikou 571158, China; zhangcaiyun-tracy@163.com; 2 Key Laboratory of Tropical Translational Medicine of Ministry of Education, Hainan Medical University, Haikou 571199, China; yongruilin@yeah.net (Y.L.); xielicui111@163.com (L.X.); w15836219306@outlook.com (Y.W.); xiezhiren166@163.com (Z.X.)

**Keywords:** *Cassytha filiformis*, aporphine alkaloids, glucose consumption

## Abstract

Aporphine alkaloids were the characteristic compounds with hypoglycemic effects in *Cassytha filiformis*. Utilizing chromatographic separation techniques including silica gel and semi-preparative high-performance liquid chromatography, three new aporphine alkaloids were successfully isolated and purified. Their structures were elucidated using various spectroscopic techniques, including one-dimensional (1D) and two-dimensional (2D) nuclear magnetic resonance (NMR) spectroscopy, as well as high-resolution electrospray ionization mass spectrometry (HRESIMS). The new compounds were identified as 10-demethylcassythine (**1**), 3-demethylcassythine (**2**), and *N*-demethyllastourvilline (**3**). The absolute configurations of the new compounds were determined using electronic circular dichroism (ECD) calculations. The effects of the new compounds on promoting glucose consumption in HepG2 cells at varying concentrations were tested. The results indicate that compound **1** significantly enhanced glucose consumption at 20 μM.

## 1. Introduction

*Cassytha filiformis* L. (Lauraceae) is a perennial stem hemi-parasitic vine—a twining herb adhering to the host plant via disk-shaped suction roots [1,2]. The plant is widely distributed in subtropical and tropical regions, including parts of Asian [3]. *C. filiformis* exhibits diverse medicinal properties, including anti-parasitic, anti-tumor, anti-diabetic, and anti-platelet aggregation activities [4,5]. Notably, it has a well-documented history of traditional use for treating diabetes in China and other regions, and chemical studies have identified alkaloids as its characteristic and bioactive constituents—a class of compounds that likely underpins its anti-diabetic potential [6]. However, the anti-diabetic constituents in this plant are still unclear. Our research interest is concentrated on the chemical compositions with unique structures and significant hypoglycemic activities from the Hainan Island, China. Aporphine alkaloids were proved to be the main compounds in *C. filiformis* [7]. Therefore, in order to find the compounds with new structures and potent anti-diabetic activities from *C. filiformis,* we focused on isolating and evaluating its alkaloidal fractions. Previously, two new aporphine alkaloids with significant glucose consumption activities were isolated and structurally characterized by our group [7]. This finding not only validated the traditional anti-diabetic usage of *C. filiformis*, but also motivated us to further explore for aporphine alkaloids with enhanced hypoglycemic effects or novel structural features. Therefore, we investigated the aporphine alkaloids in *C. filiformis* and their glucose consumption activities using a further step.

In the present study, the *C. filiformis* was extracted by extracting with ethanol under reflux then with acid dissolving and alkali precipitating to give the total alkaloid. The total alkaloid was further isolated and purified by using various chromatographic methods, including silica gel and semi-preparative HPLC, to afford three new aporphine alkaloids. The new compounds were structurally characterized by applying a combination of spectroscopic methods, including IR, UV, 1D-, 2D NMR, HRMS, etc. Specially, the stereochemistry of the new compounds were solved using the ECD method. Moreover, their promoting glucose consumption activities were evaluated on HepG2 cells to evaluate the hypoglycemic activities of these compounds. The results show that compound **1** was found to have significant glucose consumption activity at 20 μM. Using a combination of the literature and present data, a preliminary structure–activity relationship was drawn.

## 2. Results and Discussion

**Figure 1:**
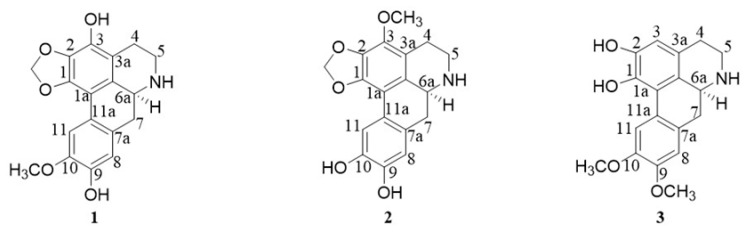
Chemical structures of compounds **1**–**3**.

The chemical analysis conducted on the alkaloid extraction fraction of *C*. *filiformis* resulted in the isolation and identification of three new aporphine alkaloids (**1**–**3**) (Figure 1).

### Structural Elucidation

**Figure 2:**
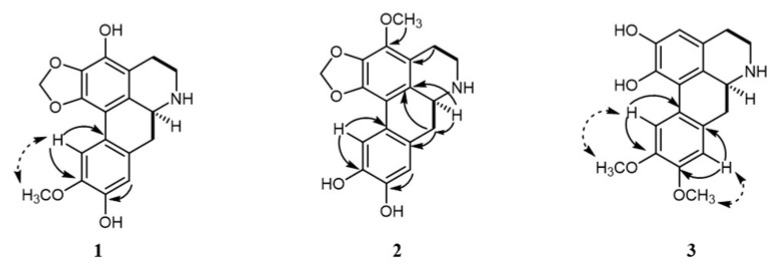
Key ^1^H-^1^H COSY (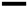), HMBC (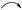), and NOESY (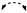) correlations of compounds **1**–**3**.

**Figure 3:**
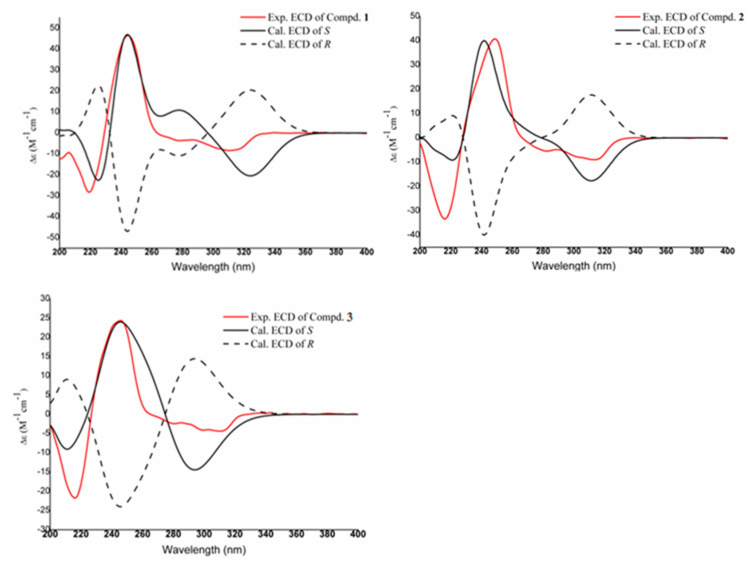
Experimental ECD and calculated ECD spectra of compounds **1**–**3** in CH_3_OH.

Compound **1** was purified as white powder with a molecular formula of C_18_H_17_NO_5_, as established from its [M + H]^+^ ion peak at *m*/*z* 328.1190 (calcd. for C_18_H_18_NO_5_ 328.1185) (Figure S2). The ^1^H NMR spectrum (Table 1) showed signals for two benzoic signals at *δ*_H_ 7.37 (^1^H, s, H-11), 6.70 (^1^H, s, H-8), one methylenedioxy at *δ*_H_ 6.12/6.00 (-OCH_2_O-), three methylene at *δ*_H_ 3.45 (^1^H, brd, *J* = 12.0 Hz, H-5β), 2.97 (^1^H, m, H-5α), 2.64 (^1^H, t, *J* = 14.0 Hz, H-7α), 2.92 (^1^H, overlapped, H-7β), 2.75 (2H, m, H-4), one methine at *δ*_H_ 4.00 (^1^H, dd, *J* = 14.0, 4.4 Hz, H-6a), and one methoxy group at *δ*_H_ 3.93 (3H, s, 3-OCH_3_). The combination of HSQC data and ^13^C-NMR data (Table 1) confirmed the presence of two benzoic groups at *δ*_C_ 145.1 (C-10), 144.3 (C-9), 143.2 (C-1), 138.6 (C-3), 135.7 (C-2), 124.3 (C-11a), 123.7 (C-1b), 121.0 (C-7a), 117.6 (C-3a), 115.7 (C-8), 114.1 (C-11), and 111.0 (C-1a). One methylenedioxy was observed at *δ*_C_ 101.0 (-OCH_2_O-). Three methylene were identified at *δ*_C_ 40.6 (C-5), 32.7 (C-7), and 21.0 (C-4), along with one methine at *δ*_C_ 52.3 (C-6a). One methoxy carbon signal was resonated at *δ*_C_ 59.4 (3-OCH_3_). The planar structure was established through ^1^H-^1^H COSY and HMBC spectral analyses (Figure 2). The ^1^H-^1^H COSY correlations from H-4 to H-5 and H-6a to H-7 allowed for the establishment of two units of C-4/C-5 and C-6a/C-7. The HMBC correlations from H-4 (*δ*_H_ 3.45) and H-5 (*δ*_H_ 2.97) to C-3a (*δ*_C_ 117.6) confirmed the connection from C-4 to C-3a. The HMBC correlations from H-7 to C-7a (*δ*_C_ 121.0) revealed linkage from C-7 to C-7a. HMBC correlations from the protons of methylenedioxy at *δ*_H_ 6.12/6.00 to C-1 (*δ*_C_ 143.2) and C-2 (*δ*_C_ 135.7) and correlation from H-5 to C-6a determined the connections of the methylenedioxy group and from C-5 to C-6a. The connections of the hydroxyl groups were determined by H-11 (*δ*_H_ 7.37) to *δ*_C_ 111.0 (C-1a), 124.3 (C-11a), 145.1 (C-10), and 144.3 (C-9), and from H-8 (*δ*_H_ 6.70) to *δ*_C_ 32.7 (C-7), 121.0 (C-7a), 145.1 (C-10), and 144.3 (C-9). The HMBC correlation from -OCH_3_ (*δ*_H_ 3.93) to C-3 (*δ*_C_ 138.6) confirmed the presence of 3-OCH_3_. Using these analyses, the NMR data of **1** was found to be similar to those of cassythine [8,9]. The differences between the two compounds focus on the substituent of C-10. In compound **1**, the substituent at C-10 is hydroxyl, while in cassythine, it is a methoxy group. The absolute configuration was further resolved by comparing experimental and calculated ECD spectra. The close match between the experimental ECD curve and the calculated spectrum of the *S* configured enantiomer unambiguously assigned the absolute configuration of 6a*S* (Figure 3). By these analyses, the structure of **1** was established and designed as 10-demethylcassythine (Figures S1–S11).

Compound **2** was purified as white powder with a molecular formula of C_18_H_17_NO_5_, as established from its [M + H]^+^ ion peak at *m*/*z* 328.1186 (calcd. for C_18_H_18_NO_5_ 328.1185). The ^1^H-NMR spectrum (Table 1) showed signals attributed to two benzoic hydrogens at *δ*_H_ 7.46 (^1^H, s, H-11), 6.78 (^1^H, s, H-8), one methylenedioxy at *δ*_H_ 6.13/6.00 (-OCH_2_O-), three methylene at *δ*_H_ 3.47 (^1^H, brd, *J* = 16.4 Hz, H-5α), 2.98 (^1^H, m, H-5β), 2.67 (^1^H, t, *J* = 12.0 Hz, H-7α), 2.98 (^1^H, brd, *J* = 12.0 Hz, H-7β), 2.76 (2H, m, H-4), one methine at *δ*_H_ 3.99 (^1^H, m, H-6a), and one methoxy group at *δ*_H_ 3.76 (3H, s, 10-OCH_3_). The ^13^C-NMR showed the existences of two benzene rings at *δ*_C_ [147.0 (C-10), 146.3 (C-9), 142.8 (C-1), 137.1 (C-3), 134.5 (C-2), 126.2 (C-1b), 122.1 (C-7a), 116.2 (C-3a), 116.0 (C-8), 111.2 (C-1a), 108.8 (C-11)], one methylenedioxy at *δ*_C_ 101.1 (-OCH_2_O-), three methylene at *δ*_c_ 41.2 (C-5), 33.3 (C-7), 21.5 (C-4), one methine carbon at *δ*_C_ 52.7, and one methoxy group at *δ*_C_ 56.4. The ^1^H-^1^H COSY correlations from H-4 to H-5 and H-6a to H-7 allowed for the establishment of two units of C-4/C-5 and C-6a/C-7. The NMR data of **1** was similar with those of cassythine [8,9]. The differences between the two compounds focus on the substituents at C-10. In compound **2**, the substituent at C-3 is hydroxyl, whereas in cassythine it is a methoxy group. This structural change was confirmed by the HMBC correlations from H-11 (*δ*_H_ 7.46) to *δ*_C_ 108.8 (C-1a), *δ*_C_ 126.2 (C-11a), and *δ*_C_ 147.0 (C-10), and from H-8 (*δ*_H_ 6.68) to *δ*_C_ 33.3 (C-7), 122.1 (C-7a), and 146.3 (C-9). The NOESY correlation between H-11 (*δ*_H_ 7.46) and 10-OCH_3_ (*δ*_H_ 3.76) verified that the location of the methoxy group was at C-10 (*δ*_C_ 56.4) (Figure 2). The absolute configuration of C-6a was determined to be *S* by comparing the experimental and calculated ECD spectrums (Figure 3). Using these analyses, the structure of **2** was established and termed as 3-demethylcassythine (Figures S13–S19).

Compound **3** was obtained as a light brown powder. Its molecular formula was deduced to be C_18_H_19_NO_4_ from its [M + H]^+^ ion peak at *m*/*z* 341.1391 (calcd. for C_18_H_20_NO_4_ 341.1392). The ^1^H-NMR spectrum of three displayed signals was attributed to three benzoic hydrogens at *δ*_H_ 8.04 (^1^H, s, H-11), 6.76 (2H, s, H-3, 8), two methoxy groups at *δ*_H_ 3.84 (3H, s, 8-OCH_3_), 3.76 (3H, s, 11-OCH_3_), three methylene at *δ*_H_ 3.58 (^1^H, m, H-5α), 3.16 (^1^H, m, H-5β), 2.74 (^1^H, t, *J* = 12.0 Hz, H-7α), 2.92 (^1^H, brd, *J* = 12.0 Hz, H-7β), 3.12 (^1^H, m, H-4), 2.83 (^1^H, m, H-4), and one methine at *δ*_H_ 4.14 (^1^H, d, *J* = 12.0 Hz, H-6a). The ^13^C-NMR shows the existences of two benzene rings [148.4 (C-10), 146.5 (C-9), 146.4 (C-2), 142.1 (C-1), 126.9 (C-3a), 123.5 (C-1b), 121.8 (C-7a), 121.5 (C-11a), 120.1 (C-1a), 115.6 (C-8), 114.2 (C-11), and 110.1 (C-3)], three methylene at *δ*_C_ 41.0 (C-5), 32.9 (C-7), 25.2 (C-4), one methine carbon at *δ*_C_ 52.6, and two methoxy groups at *δ*_C_ 56.5 and 56.4. These NMR data were similar to those of lastourvilline, except for the absence of *N*-methyl [10]. This conclusion was confirmed by the relevant HSQC and HMBC correlations. The two methoxy groups attached at C-9 and C-10 were also verified by the NOESY correlations between *δ*_H_ 3.84 (3H, s, 8-OCH_3_) and *δ*_H_ 6.76 (H-8), 3.76 (3H, s, 11-OCH_3_), and *δ*_H_ 8.04 (H-11) (Figure 2). The absolute configuration of C-6a was also determined to be *S* by comparing the experimental CD curve and the calculated spectrum ECD (Figure 3). Therefore, the structure of **3** was determined and termed as *N*-demethyllastourvilline (Figures S20–S28).

**Figure 4:**
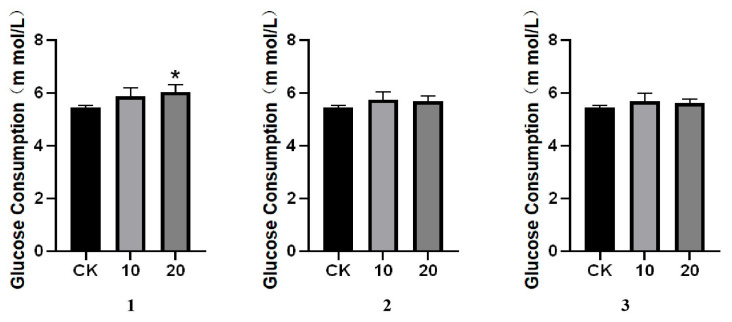
Effects of compounds **1**–**3** on promoting glucose consumption on HepG2 cells. All data are expressed as means ± SD, * *p* < 0.05 compared with control (CK).

The new compounds were tested for their glucose consumption promoting activities. The results showed that compound **1** exhibited significant activity at the dosage of 20 μM. According to the results, the methyl group at different positions affected the ability on promoting the glucose consumption (Figure 4).

## 3. Materials and Methods

### 3.1. General

Optical rotations were obtained with a JASCO P-2200 polarimeter (Hachioji, Japan). UV spectra were recorded on a JASCO V-650 spectrometer (JASCO INTERNATIONAL Co., Ltd., Tokyo, Japan). ECD spectra were obtained on a JASCO J-810 spectrometer (JASCO INTERNATIONAL Co., Ltd., Tokyo, Japan). One-dimensional (^1^H and ^13^C) NMR and two-dimensional (^1^H–^1^H COSY, HSQC, HMBC, and NOESY) NMR experiments were performed on an AVANCE III HD 400 MHz spectrometer (Fällanden, Switzerland) operating at 400 MHz for ^1^H and 100 MHz for ^13^C NMR, respectively. Chemical shifts were expressed in *δ* (ppm) and coupling constants in Hz. HRESIMS spectra were acquired using the Waters Xevo G2-XS Q-TOF mass spectrometer (Milford, MA, USA). High-performance liquid chromatography (HPLC) was performed on a Shimadzu Liquid Chromatography LC-16 system (Kyoto, Japan) using a column of Agilent (Santa Clara, CA, USA, ZORBAX SB-Phenyl, 2.5 mm × 250 mm, 5 µm) equipped with an SPD-16 detector (UV/VIS, Shimadzu). A mixture of acetonitrile: 0.1% Phosphoric acid-H_2_O was used as eluent. HepG2 cells were purchased from the Institute of Cell Biology (SNL-083), Chinese Academy of Sciences (Shanghai, China). The DMEM medium, fetal bovine serum (FBS), trypsin, antibiotics, and phosphate-buffer solution were purchased from Gibco-Invitrogen (Grand Island, NY, USA). A glucose assay kit (Glucose Oxidase Method) was purchased from BSBE Biotech Co., LLC (Beijing, China).

### 3.2. Plant Material

The whole herb of *C. filiformis* was collected from Guangxi Province, China, in July 2023. The plant was identified by Professor Yuguang Fan of Hainan Medical University, and a voucher specimen has been deposited at the herbarium of the School of Pharmaceutical Science, Hainan Medical University (No. CF202307).

### 3.3. Extraction and Isolation

The dried and powdered *C. filiformis* (50 kg) was extracted under soak by seven times with 95% ethanol for 24 h each time. The crude extract was vacuum-evaporated to recover the solvent until the odor of ethanol disappeared at 60 °C. The pH value of the crude extract was adjusted to about 2 by adding 1% HCl. The dichloromethane was added to the acidic solvent to give the sediment. Then the sediment was dissolved in 1% HCl and filtered for five times to obtain the solvent. The pH value of the solvent was adjusted to about 10 by adding ammonia and then extracted by dichloromethane for three times. The extract was concentrated to give a total crude alkaloid (264.0 g). An aliquot (226.0 g) was subjected to column chromatography and eluted with a gradient of CH_2_Cl_2_-CH_3_OH (50:1, 19:1, 9:1, 4:1, 2:1, 1:1, *v*/*v*) and concentrated under reduced pressure. The fractions were collected based on volume, with each gradient being about 10.0 L eluent. Finally, nine fractions (A–I) were acquired. Fraction B was purified by semi-preparative HPLC eluting with acetonitrile: 0.1% Phosphoric acid-H_2_O (2.0 mL/min) to give **1** (12.5 mg) and **2** (21.0 mg). Fraction C was purified by semi-preparative HPLC eluting with acetonitrile: 0.1% Phosphoric acid-H_2_O (2.0 mL/min) to give **3** (12.2 mg).

### 3.4. Glucose Consumption Assay

HepG2 cells were seeded at 1 × 10^5^ cells/mL cells in BeyoGold™ 96 well cell culture plates (Beyotime, Shanghai, China). After 24 h, the original culture medium was removed and then the culture plate was filled with the culture medium containing the drug (0.1% DMSO, 0.5% FBS medium). The required culture medium when preparing the medicine (DMEM + 0.5% FBS + 1% P/S). The experiment was divided into a control group (CK) and groups treated with different concentrations of the test drug. Each group had six replicates, and the cells were cultured in an incubator for 24 h. The culture plates were taken out, and 2 µL of cell supernatant from each well in the culture plates was transferred to a new plate for grouping and labeling. At the same time, an empty group was created in the new culture plates. In total, 2 µL of standard medium was set up in six replicates, and 200 µL of glucose detection reagent was added to each well. The mixture was mixed evenly using a 96-well plate shaker and incubated at 37 °C for 10 min. The absorbance value was measured at a wavelength of 505 nm. The experiment was independently repeated three times. Calculation: glucose consumption concentration = initial glucose concentration − (initial glucose concentration × OD value of test drug/OD value of standard solution) [11,12].

### 3.5. ECD Calculation

ECD spectra were obtained at room temperature on a Jasco J-815 CD spectrometer, flushed with dry nitrogen using 0.1 cm and 1 cm quartz cuvettes adopting the following conditions: 100 nm/min scanning rate, 2 nm resolution and 32 scans. Samples were dissolved in methanol at 10^−4^ M or 10^−3^ M concentrations, and spectra were solvent subtracted. The ECD calculations were carried out according to previously reported methods [13].

### 3.6. Spectroscopic Data of the New Compounds

#### 3.6.1. 3-Demethylcassythine

White powder; [α]_D_ = +21.6 (c 0.1, MeOH); UV (MeOH) *λ_max_* (*logε*) 222 (1.22), 286 (0.76) nm; ^1^H NMR (400 MHz, DMSO-*d*_6_), ^13^C NMR (100 MHz, DMSO-*d*_6_) (see Table 1); HRESIMS: *m*/*z* 328.1186 (calcd. for C_18_H_18_NO_5_ 328.1185).

#### 3.6.2. 10-Demethylcassythine

White powder; [α]_D_ = +19.5 (c 0.1, MeOH); UV (MeOH) *λ_max_* (*logε*) 220 (1.18), 285 (0.73) nm; ^1^H NMR (400 MHz, DMSO-*d*_6_), ^13^C NMR (100 MHz, DMSO-*d*_6_) (see Table 1); HRESIMS: *m*/*z* 328.1186 (calcd. for C_18_H_18_NO_5_ 328.1185).

#### 3.6.3. *N*-Demethyllastourvilline

Light brown powder; [α]_D_ = +16.7 (c 0.1, MeOH); UV (MeOH) *λ_max_* (*logε*) 217 (0.82), 280 (0.20) nm; ^1^H NMR (400 MHz, DMSO-*d*_6_), ^13^C NMR (100 MHz, DMSO-*d*_6_) (see Table 1); HRESIMS: *m*/z 341.1391 (calcd. for C_18_H_20_NO_4_ 341.1392).

## 4. Conclusions

Three new aporphine alkaloids were obtained and structurally characterized by applying spectroscopic data. Their stereochemistry of C-6a were also solved by applying the ECD method. Conformational analyses were carried out via random searching in Sybyl-X 2.0 using the MMFF94S force field with an energy cutoff of 5 kcal/mol [14]. The results show the three lowest energy conformers. Subsequently, geometry optimizations and frequency analyses were implemented at the B3LYP-D3(BJ)/6-31G* level in CPCM methanol using ORCA5.0.3 [15]. All conformers used for property calculations in this work were characterized to be stable points on potential energy surface (PES) with no imaginary frequencies. The excitation energies, oscillator strengths, and rotational strengths (velocity) of the first 60 excited states were calculated using the TD-DFT methodology at the PBE0/def2-TZVP level in CPCM methanol using ORCA5.0.3 [15]. The ECD spectra were simulated by the overlapping Gaussian functions (half the bandwidth at 1/e peak height, sigma = 0.30 for all) [16]. Gibbs-free energies for conformers were determined by using thermal correction at the B3LYP-D3(BJ)/6-31G* level and electronic energies evaluated at the wB97M-V/def2-TZVP level in CPCM methanol using ORCA5.0.3. To obtain the final spectra [15], the simulated spectra of the conformers were averaged according to the Boltzmann distribution theory and their relative Gibbs free energies (∆G). By comparing the experiment spectra with the calculated model molecules, the absolute configuration of the only chiral center was determined to be *S*.

The glucose consumption ability of patients with diabetes is influenced by insulin function, and the decline in this consumption ability is an important aspect of the pathophysiological process of diabetes [17,18]. Due to insulin issues, cells in diabetic patients cannot effectively utilize glucose in the blood, leading to a decrease in glucose consumption capacity [19]. Moreover, cells may also fail to function properly due to a lack of energy [20]. Therefore, compounds that could promote glucose consumption are important candidates for new drug discovery. In the present study, three new aporphine alkaloids were isolated and identified from *C*. *filiformis*. These compounds were tested for their glucose consumption enhancing activities. A structure–activity relationship of these compounds could be drawn preliminarily. Combined with other aporphine alkaloids with glucose lowering effects reported by our research group [7,21], the positions of the methoxy substituents on the benzene rings affected the activities. Previously, laurolitsine was found to have the ability of glucose lowering effects both in vitro and in vivo by our group. The three compounds shared the same carbon skeleton, but with different substituents on the benzenes rings. Laurolitsine displayed hypoglycemic activity with the dosage at 2.5 μM in vivo while compound **1** was 20 μM. Another two new aporphine alkaloids showed better activities at dosage of 0.3125 and 6.35 μM, respectively [7]. Therefore, the number and positions of substituents on the benzene rings significantly affected the activities. It is noteworthy that the glucose consumption activity of compound 1 was observed at a concentration of 20 μM. We acknowledge that this concentration, while common in initial bioactivity screening, might elicit non-specific effects on cellular metabolism or gene expression. Therefore, the activity observed at this concentration should be interpreted with caution and warrants further investigation at lower, more physiologically relevant doses to confirm its specificity and mechanisms. However, a detailed structure–activity relationship can not be drawn because of limited reported compounds. Apart from our reports, natural aporphine alkaloids have been reported for possessing significant hypoglycemic activities [22,23]. For example, nuciferine elicited anti-diabetic activity and was regarded as a potentially diabetic adjuvant agent or food additive [24]. Magnofluorine, another aporphine alkaloid obtained from natural plants, was verified to have the ability of lowering blood glucose and improve the complications of diabetes [25]. Therefore, aporphine alkaloids with glucose promoting activities are important sources of lead compounds for treating diabetes and its related complications [26]. This study provides new perspectives on the structure and activity of aporphine alkaloids from *C. filiformis*. Building on these findings, in our future work, we will expand the tested concentration ranges and employ transcriptomic or metabolomic analyses to precisely elucidate the mechanisms of action, thereby deepening the exploration of their anti-diabetic potential. The unique structural characteristics of the aporphine alkaloids and the underlying targets are essential factors for new drug discovery [27,28]. For further studies, more aporphine alkaloids with special structures and underlying mechanisms should be investigated in depth.

## Figures and Tables

**Table 1:** NMR data of compounds **1**–**3** (*δ* in ppm, *J* values in Hz).

No.	Compound 1		Compound 2		Compound 3	
	*δ*_C_	*δ*_H_	*δ*_C_	*δ*_H_	*δ*_C_	*δ*_H_
1	143.2		142.8		142.1	
1a	111.0		111.2		120.1	
1b	123.7		126.2		123.5	
2	135.7		134.5		146.4	
3	138.6		137.1		110.1	
3a	117.6		116.2		126.9	
4	21.0	2.75, m	21.5	2.76, m	25.2	3.12, m; 2.83, m
5α	40.6	3.45, brd, 12.0	41.2	3.47, brd, 16.4	41.0	3.58, m
5β	40.6	2.97, m	41.2	2.98, m	41.0	3.16, m
6a	52.3	4.00, dd,14.0, 4.4	52.7	3.99, m	52.6	4.14, d, 12.0
7α	32.7	2.64, t, 14.0	33.7	2.67, d, 12.4	32.9	2.74, t, 12.0
7β	32.7	2.92, overlapped	33.7	2.98, d, 12.4	32.9	2.92, d, 12.0
7a	121.0		122.1		121.8	
8	115.7	6.70, s	116.0	6.78, s	115.6	
9	144.3		146.3		146.5	
10	145.1		147.0		148.4	
11	114.1	7.37, s	108.8	7.46, s	114.2	8.04, s
11a	124.3		124.0		121.5	
3-OMe	59.4	3.93, s				
8-OMe					56.4	3.84, s
10-OMe			56.4	3.76, s		
11-OMe					56.5	3.76, s
O-CH_2_-O	101.1	6.12, s; 6.00, s	101.1	6.13, s; 6.00, s		

Measured at 400 MHz for ^1^H NMR and 100 MHz for ^13^C NMR in DMSO-*d*_6_.

## Data Availability

The original contributions presented in this study are included in the article/Supplementary Materials. Further inquiries can be directed to the corresponding authors.

## Supplementary Materials

The following supporting information can be downloaded at https://www.mdpi.com/article/10.3390/molecules30234544/s1. Figure S1. UV-Vis Absorption Spectrum of 10-Demethylcassythine. Figure S2. Mass Spectrum of 10-Demethylcassythine. Figure S3. ^1^H NMR Spectrum of 10-Demethylcassythine. Figure S4. ^1^H NMR Spectrum of 10-Demethylcassythine (Expansion 1). Figure S5. ^1^H NMR Spectrum of 10-Demethylcassythine (Expansion 2). Figure S6. ^13^C NMR Spectrum of 10-Demethylcassythine. Figure S7. DEPT Spectrum of 10-Demethylcassythine. Figure S8. ^1^H-^1^H COSY Spectrum of 10-Demethylcassythine. Figure S9. HSQC Spectrum of 10-Demethylcassythine. Figure S10. HMBC Spectrum of 10-Demethylcassythine. Figure S11. NOESY Spectrum of 10-Demethylcassythine. Figure S12. Experimental and Calculated ECD Spectra of 10-Demethylcassythine. Figure S13. UV-Vis Absorption Spectrum of 3-Demethylcassythine. Figure S14. Mass Spectrum of 3-Demethylcassythine. Figure S15. ^1^H NMR Spectrum of 3-Demethylcassythine. Figure S16. ^13^C NMR Spectrum of 3-Demethylcassythine. Figure S17. ^1^H-^1^H COSY Spectrum of 3-Demethylcassythine. Figure S18. HSQC Spectrum of 3-Demethylcassythine. Figure S19. HMBC Spectrum of 3-Demethylcassythine. Figure S20. NOESY Spectrum of 3-Demethylcassythine. Figure S21. Experimental and Calculated ECD Spectra of 3-Demethylcassythine. Figure S22. UV-Vis Absorption Spectrum of *N*-demethyl. Figure S23. Mass Spectrum of *N*-demethyl. Figure S24. ^1^H NMR Spectrum of *N*-demethyl. Figure S25. ^13^C NMR Spectrum of *N*-demethyl. Figure S26. DEPT Spectrum of *N*-demethyl. Figure S27. HSQC Spectrum of *N*-demethyl. Figure S28. HMBC Spectrum of *N*-demethyl. Figure S29. ^1^H-^1^H COSY Spectrum of *N*-demethyl. Figure S30. NOESY Spectrum of *N*-demethyl. Figure S31. Experimental and Calculated ECD Spectra of *N*-demethyl.

## References

[1] Rosli R., Tennakoon K.U., Yaakub M.Y.S.M., Zainal Ariffin N.A.H., Metali F. (2024). Host selectivity and distribution of *Cassytha filiformis* in the coastal bornean heath forests. Trop. Life Sci. Res..

[2] Li Q., Liu X., Liu K., Ren H., Jian S., Lu H., Cheng Y., Zou Q., Huang Y. (2025). The invasion of *Cassytha filiformis* accelerated the litter decomposition of native plant communities in small tropical coral islands. BMC Plant Biol..

[3] Cheung W.L., Law C.Y., Lee H.C.H., Tang C.O., Lam Y.H., Ng S.W., Chan S.S., Chow T.C., Pang K.S., Mak T.W.L. (2018). Gelsemium poisoning mediated by the non-toxic plant *Cassytha filiformis* parasitizing *Gelsemium elegans*. Toxicon.

[4] Agbodjento E., Klotoé J.R., Sacramento T.I., Dougnon T.V., Déguenon E., Agbankpé J., Fabiyi K., Assogba P., Hounkanrin M.P., Akotegnon R. (2020). Larval cytotoxic and subacute toxicity of Gardenia ternifolia, Rourea coccinea, and *Cassytha filiformis* used in Traditional Medicine of Benin (West Africa). J. Toxicol..

[5] Nasrollahzadeh M., Issaabadi Z., Sajadi S.M. (2018). Green synthesis of a Cu/MgO nanocomposite by *Cassytha filiformis* L. extract and investigation of its catalytic activity in the reduction of methylene blue, congo red and nitro compounds in aqueous media. RSC Adv..

[6] Ezuruike U.F., Chieli E., Prieto J.M. (2019). In vitro modulation of glibenclamide transport by P-glycoprotein inhibitory antidiabetic African plant extracts. Planta Med..

[7] Huang Z., Cao M.Y., Wang R.Q., Zhang Y., Zhang X.P., Dong L., Zhang C.Y. (2022). Two new aporphine alkaloids with glucose consumption increasing activity from *Cassytha filiformis*. Phyt. Lett..

[8] Huang Z., Chen M., Zhang Y., Zhang X., Dong L., Zhang C. (2023). Analysis of aporphine alkaloids in *Cassytha filiformis*. J. Trop. Biol..

[9] Stévigny C., Block S., De Pauw-Gillet M.C., De Hoffmann E., Llabrès G., Adjakidjé V., Quetin-Leclercq J. (2002). Cytotoxic aporphine alkaloids from *Cassytha filiformis*. Planta Med..

[10] Jacqueline E.R., Jacques B., David Z.S. (1985). Isolation of a new alkaloid from *Artabotrys lastourvillensis*. J. Nat. Prod..

[11] Wang S., Wu C., Li X., Zhou Y., Zhang Q., Ma F., Wei J., Zhang X., Guo P. (2017). Syringaresinol-4-O-β-d-glucoside alters lipid and glucose metabolism in HepG2 cells and C2C12 myotubes. Acta Pharm. Sin. B.

[12] Zhao S.L., Liu D., Ding L.Q., Liu G.K., Yao T., Wu L.L., Li G., Cao S.J., Qiu F., Kang N. (2024). Schisandra chinensis lignans improve insulin resistance by targeting TLR4 and activating IRS-1/PI3K/AKT and NF-κB signaling pathways. Int. Immunopharmacol..

[13] Du C., Li X., Chen J., Luo L., Yuan C., Yang J., Hao X., Gu W. (2024). Discovery of Coumarins from *Zanthoxylum dimorphophyllum* var. *spinifoliumas* and Their Potential against Rheumatoid Arthritis. Molecules.

[14] Bhujbal S.P., Keretsu S., Cho S.J. (2021). Molecular modelling studies on pyrazole derivatives for the design of potent rearranged during transfection kinase inhibitors. Molecules.

[15] Neese F. (2018). Software update: The ORCA program system, version 4.0. WIREs Comput. Mol. Sci..

[16] Stephens P.J., Harada N. (2010). ECD cotton effect approximated by the Gaussian curve and other methods. Chirality.

[17] Kalyani R.R., Neumiller J.J., Maruthur N.M., Wexler D.J. (2025). Diagnosis and treatment of Type 2 diabetes in adults: A review. JAMA.

[18] Harris S. (2025). Nutrition and diet in type 2 diabetes management. Br. J. Nurs..

[19] Panwar A., Malik S.O., Adib M., Lopaschuk G.D. (2025). Cardiac energy metabolism in diabetes: Emerging therapeutic targets and clinical implications. Am. J. Physiol. Heart Circ. Physiol..

[20] Gower B.A., Goss A.M., Yurchishin M.L., Deemer S.E., Sunil B., Garvey W.T. (2025). Effects of a Carbohydrate-Restricted Diet on beta-Cell Response in Adults with Type 2 Diabetes. J. Clin. Endocrinol. Metab..

[21] Zhang Y., Wang R.Q., Yang Y.N., Ma N., Zhou Z., Tan Y.F., Dong L., Li Y.Y., Lu W.Y., Wu C.M. (2022). Laurolitsine ameliorates type 2 diabetes by regulating the hepatic LKB1-AMPK pathway and gut microbiota. Phytomedicine.

[22] Amssayef A., Eddouks M. (2023). Alkaloids as promising agents for the management of insulin resistance: A review. Curr. Pharm. Des..

[23] Teerapongpisan P., Suthiphasilp V., Kumboonma P., Maneerat T., Duangyod T., Charoensup R., Promnart P., Laphookhieo S. (2024). Aporphine alkaloids and a naphthoquinone derivative from the leaves of *Phaeanthus lucidus* Oliv. and their alpha-glucosidase inhibitory activity. Phytochemistry.

[24] Wang F.X., Zhu N., Zhou F., Lin D.X. (2021). Natural aporphine alkaloids with potential to impact metabolic syndrome. Molecules.

[25] Wan Y., Xia J., Xu J.F., Chen L., Yang Y., Wu J.J., Tang F., Ao H., Peng C. (2022). Nuciferine, an active ingredient derived from lotus leaf, lights up the way for the potential treatment of obesity and obesity-related diseases. Pharmacol. Res..

[26] Durmaz L., Kiziltas H., Guven L., Karagecili H., Alwasel S., Gulcin İ. (2022). Antioxidant, antidiabetic, anticholinergic, and antiglaucoma effects of magnofluorine. Molecules.

[27] Sun J., Zhan X., Wang W., Yang X., Liu Y., Yang H., Deng J., Yang H. (2024). Natural aporphine alkaloids: A comprehensive review of phytochemistry, pharmacokinetics, anticancer activities, and clinical application. J. Adv. Res..

[28] Zhu R., Jiang G., Tang W., Zhao X., Chen F., Zhang X., Ye N. (2023). Aporphines: A privileged scaffold in CNS drug discovery. Eur. J. Med. Chem..

