# Supplementary material for: Three New Aporphine Alkaloids with Glucose Consumption Increase Activity from Cassytha filiformis
Source: Molecules. 2025 Nov 25;30(23):4544. doi: 10.3390/molecules30234544 (PMC12692773; doi:10.3390/molecules30234544)
Supplement: Supplementary file 1 [file molecules-30-04544-s001.zip › molecules-3925215-supplementary.pdf]

molecules

Supplementary materials

Three New Aporphine Alkaloids with Glucose Consumption Increase Activity from *Cassytha filiformis*

Caiyun Zhang [1,2,†], Yongrui Lin [2,†], Licui Xie [2], Yiru Wang [2], Zhiren Xie [2], Lin Dong [2,*] and Yanhui Fu [1,*]

[1] College of Chemistry and Chemical Engineering, Hainan Normal University, Haikou 571158, China; zhangcaiyun-tracy@163.com
[2] Key Laboratory of Tropical Translational Medicine of Ministry of Education, Hainan Medical University, Haikou 571199, China; yongruilin@yeah.net (Y.L.); xielicui111@163.com (L.X.); w15836219306@outlook.com (Y.W.); xiezhiren166@163.com (Z.X.)
* Correspondence: hy0207014@muhn.edu.cn (L.D.); fuyanhui80@163.com (Y.F.)
† These authors have contributed equally to this work.

Abstract

Aporphine alkaloids were the characteristic compounds with hypoglycemic effects in *Cassytha filiformis*. Utilizing chromatographic separation techniques including silica gel and semi-preparative high-performance liquid chromatography, three new aporphine alkaloids were successfully isolated and purified. Their structures were elucidated using various spectroscopic techniques, including one-dimensional (1D) and two-dimensional (2D) nuclear magnetic resonance (NMR) spectroscopy, as well as high-resolution electrospray ionization mass spectrometry (HRESIMS). The new compounds were identified as 10-demethylcassythine (**1**), 3-demethylcassythine (**2**), and *N*-demethyllastourvilline (**3**). The absolute configurations of the new compounds were determined using electronic circular dichroism (ECD) calculations. The effects of the new compounds on promoting glucose consumption in HepG2 cells at varying concentrations were tested. The results indicate that compound **1** significantly enhanced glucose consumption at 20 μM.

Keywords: *Cassytha filiformis*; aporphine alkaloids; glucose consumption

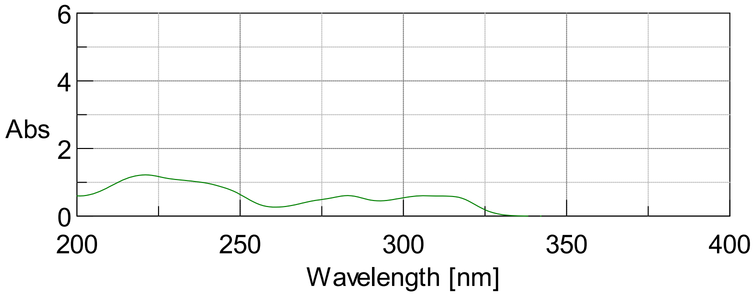

Figure S1. UV-Vis Absorption Spectrum of 10-Demethylcassythine

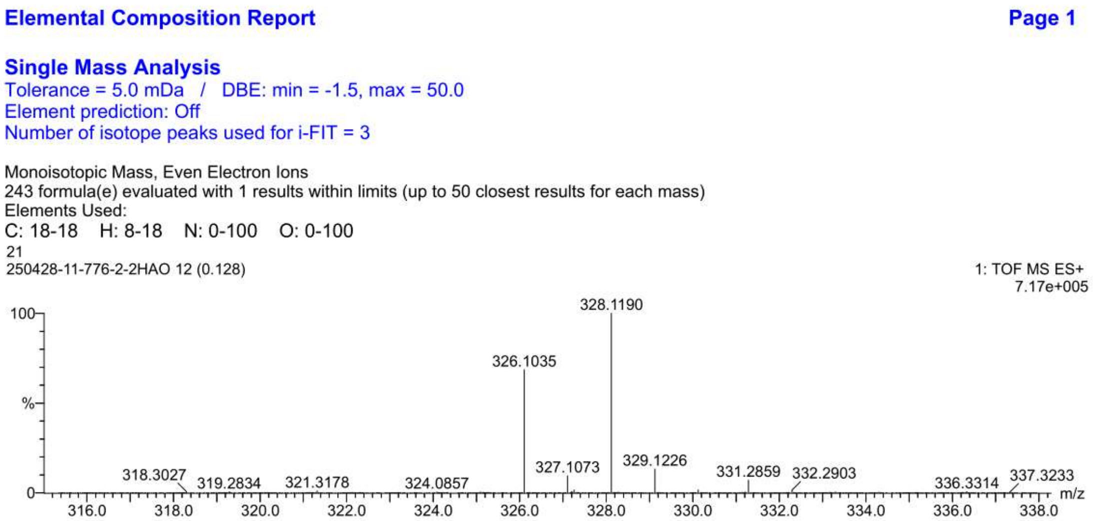

Figure S2. Mass Spectrum of 10-Demethylcassythine

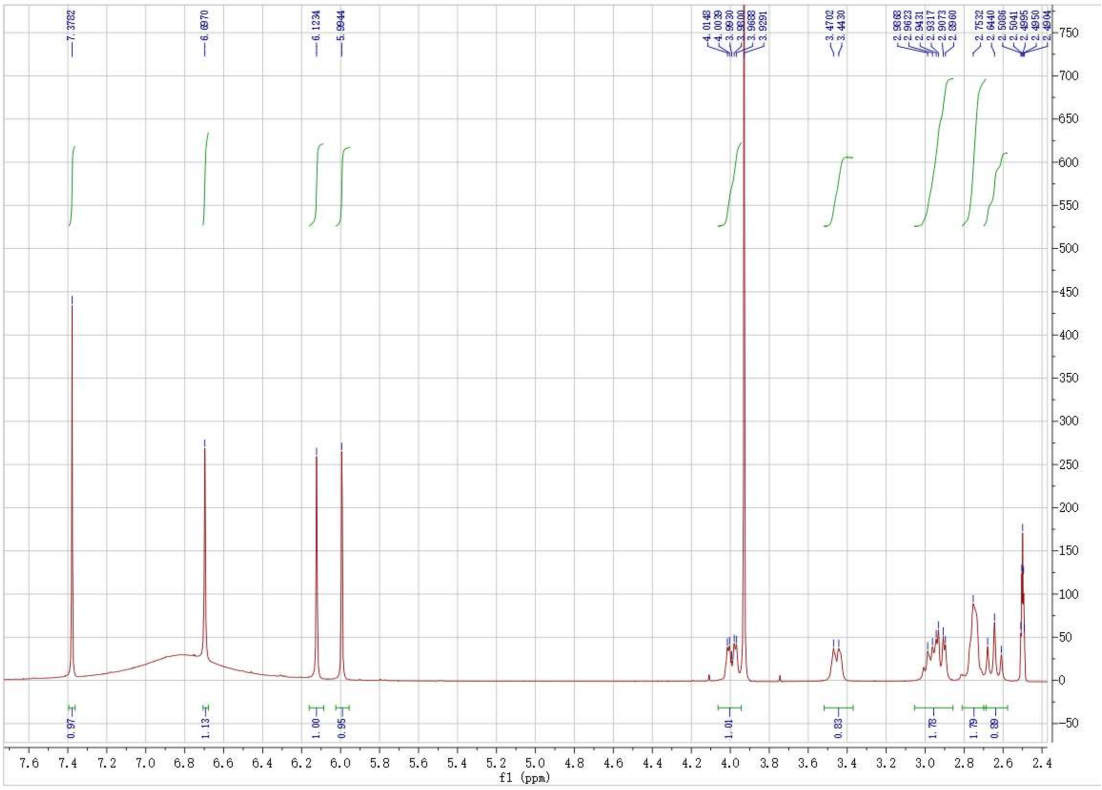

Figure S3. ^{1}H NMR Spectrum of 10-Demethylcassythine

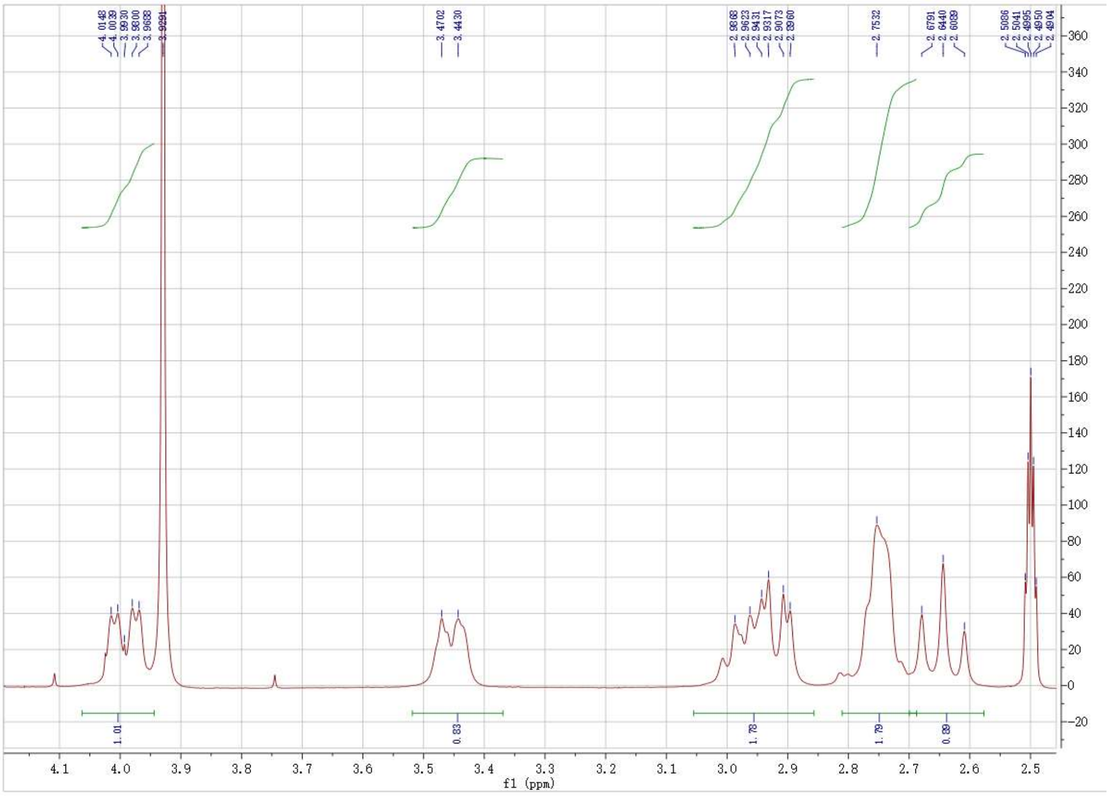

Figure S4. ¹H NMR Spectrum of 10-Demethylcassythine (Expansion 1)

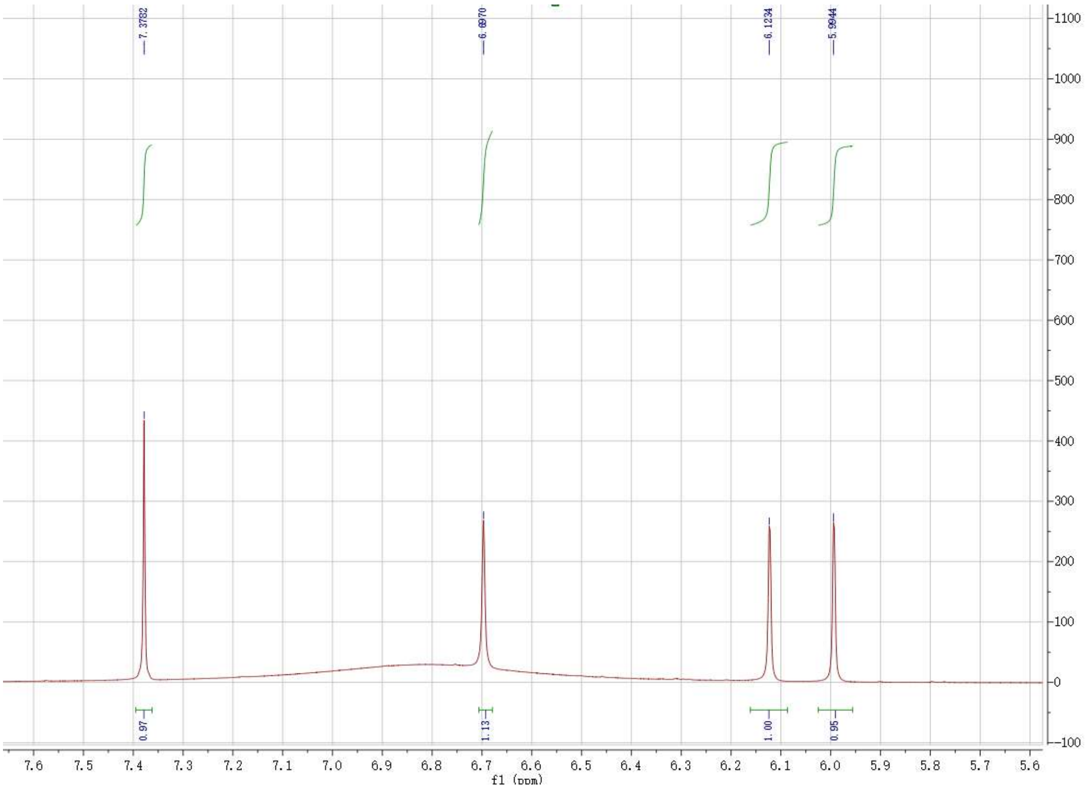

Figure S5. ¹H NMR Spectrum of 10-Demethylcassythine (Expansion 2)

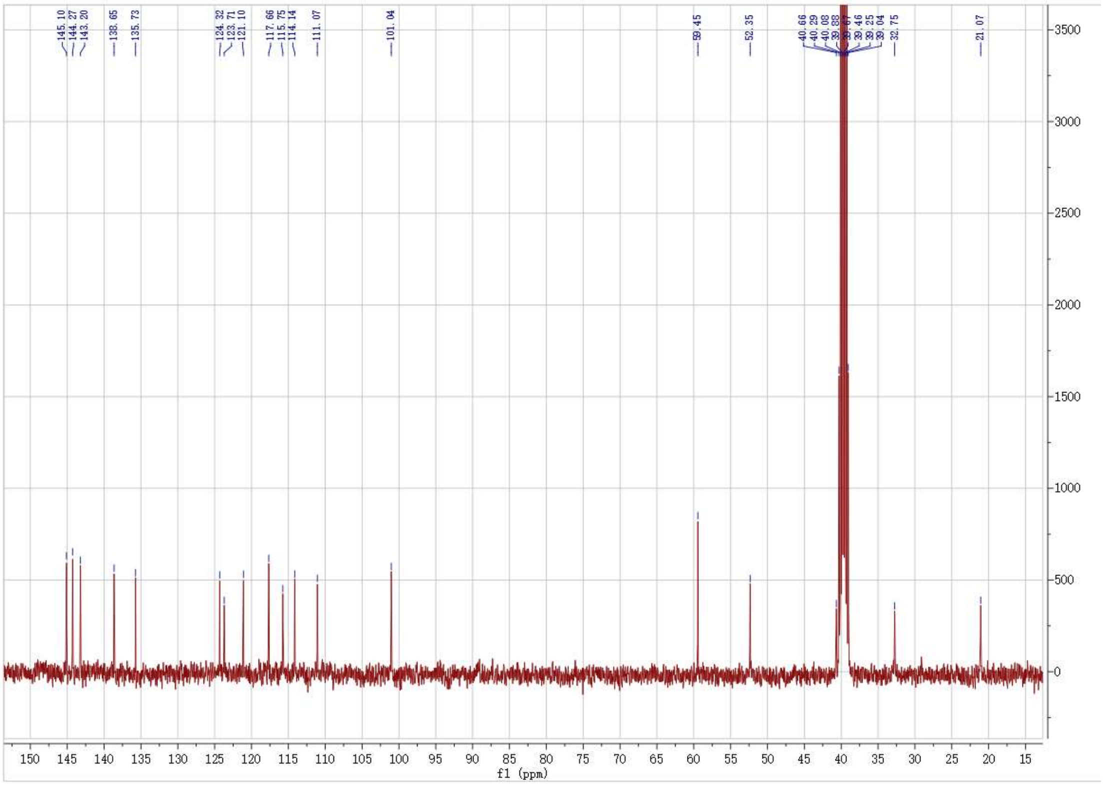

Figure S6. ^{13}C NMR Spectrum of 10-Demethylcassythine

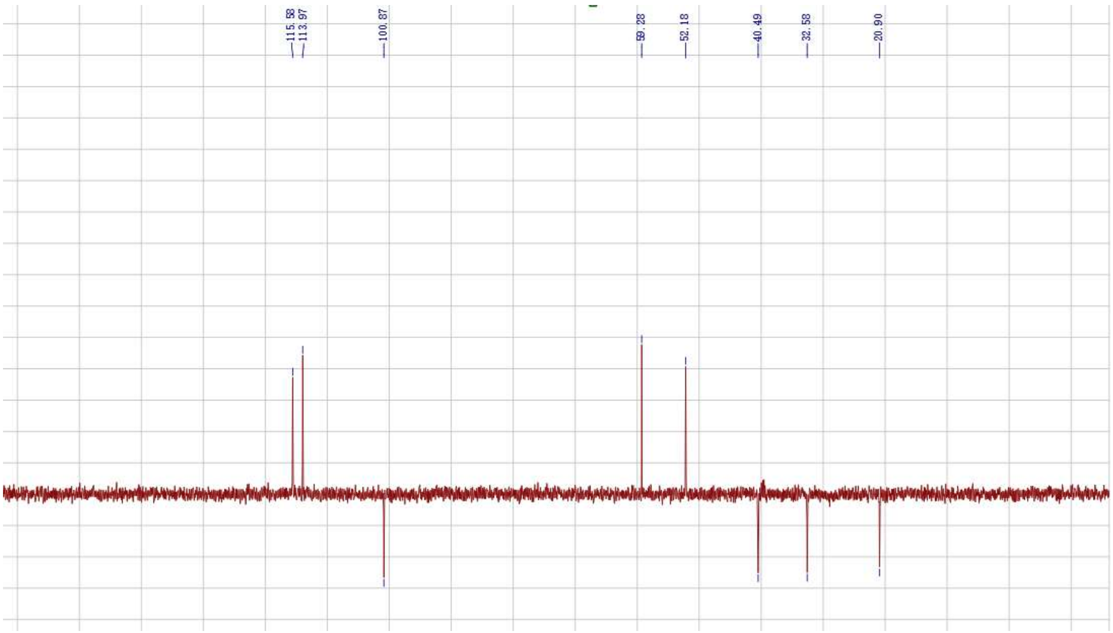

Figure S7. DEPT Spectrum of 10-Demethylcassythine

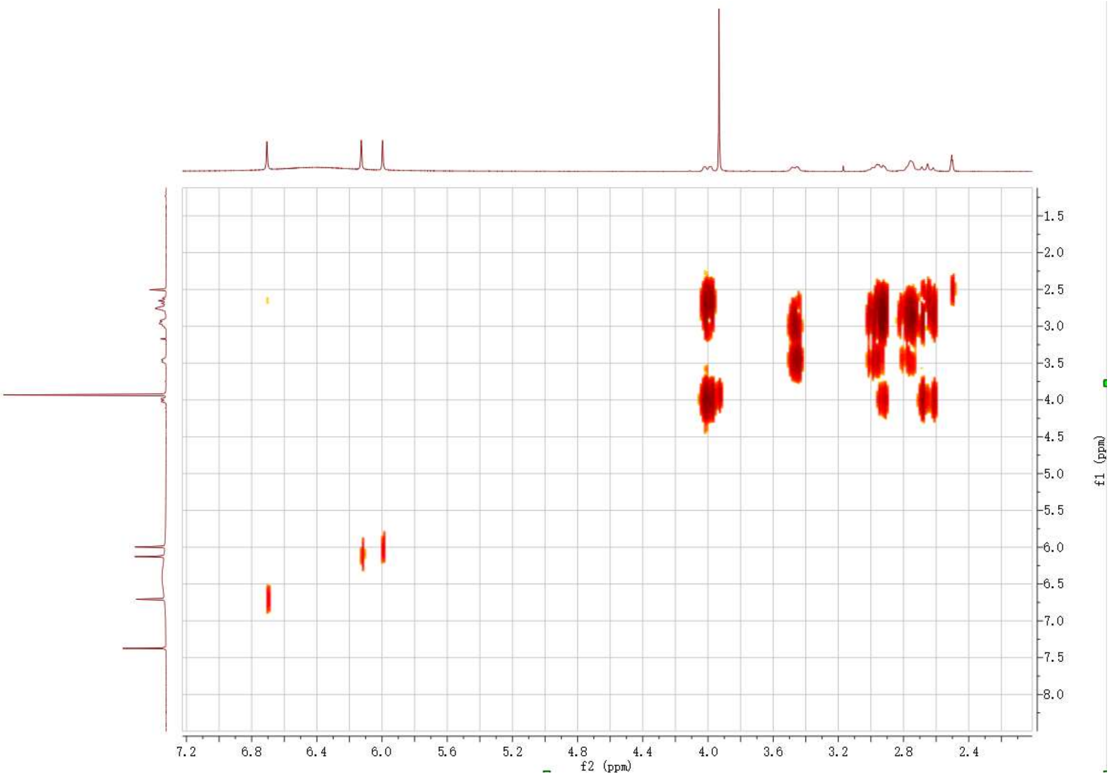

Figure S8. ^{1}H-^{1}H COSY Spectrum of 10-Demethylcassythine

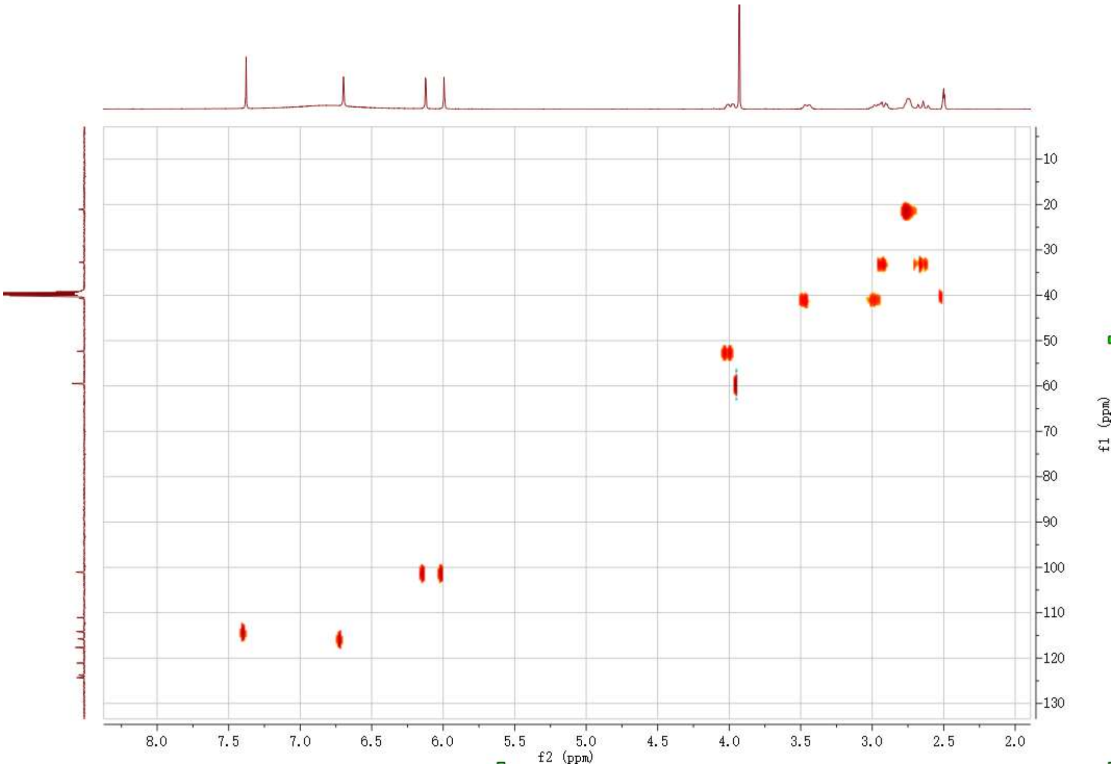

Figure S9. HSQC Spectrum of 10-Demethylcassythine

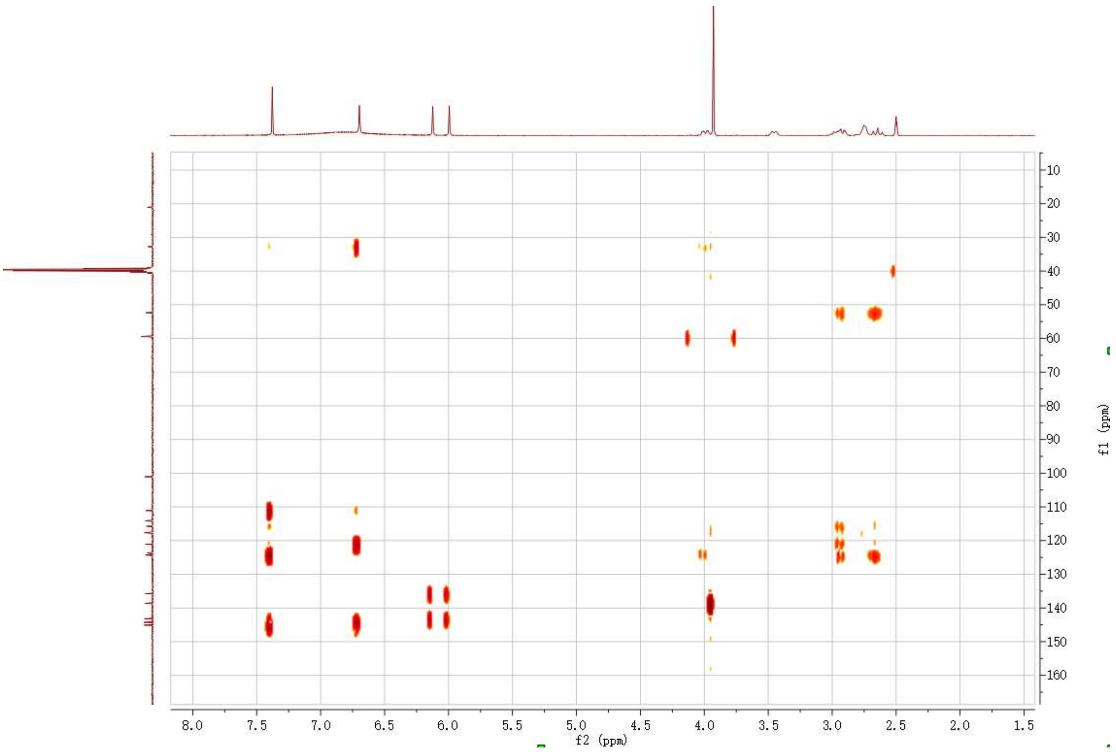

Figure S10. HMBC Spectrum of 10-Demethylcassythine

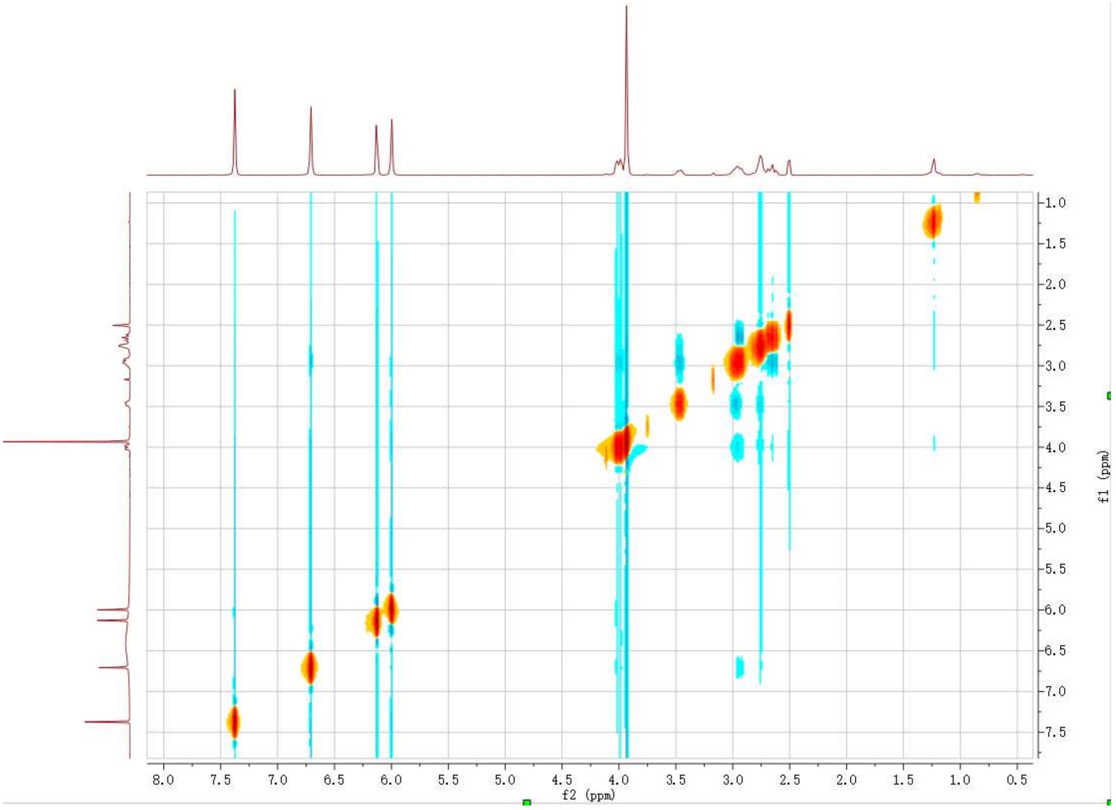

Figure S11. NOESY Spectrum of 10-Demethylcassythine

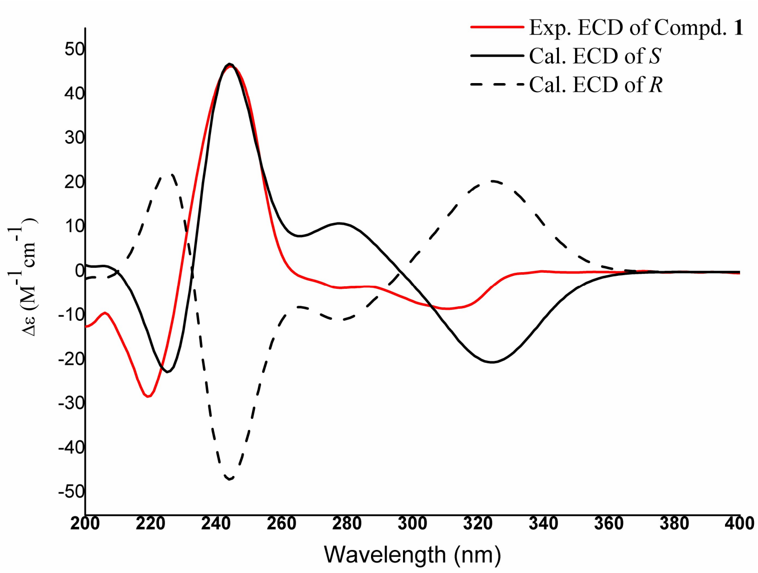

Figure S12. Experimental and Calculated ECD Spectra of 10-Demethylcassythine

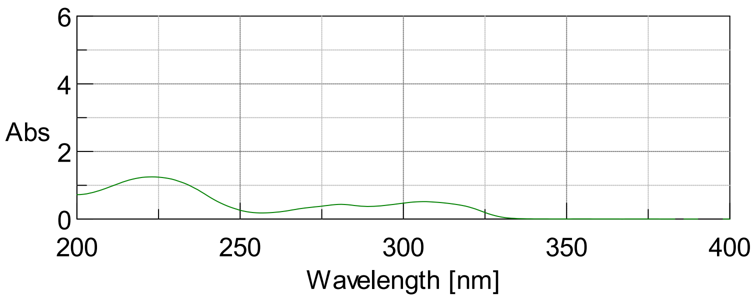

Figure S13. UV-Vis Absorption Spectrum of 3-Demethylcassythine

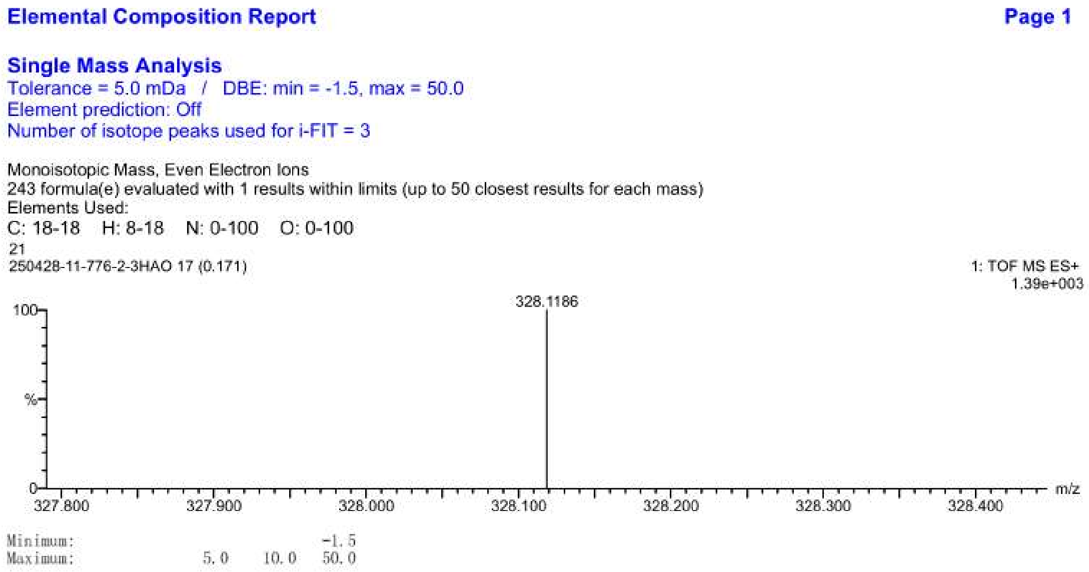

Figure S14. Mass Spectrum of 3-Demethylcassythine

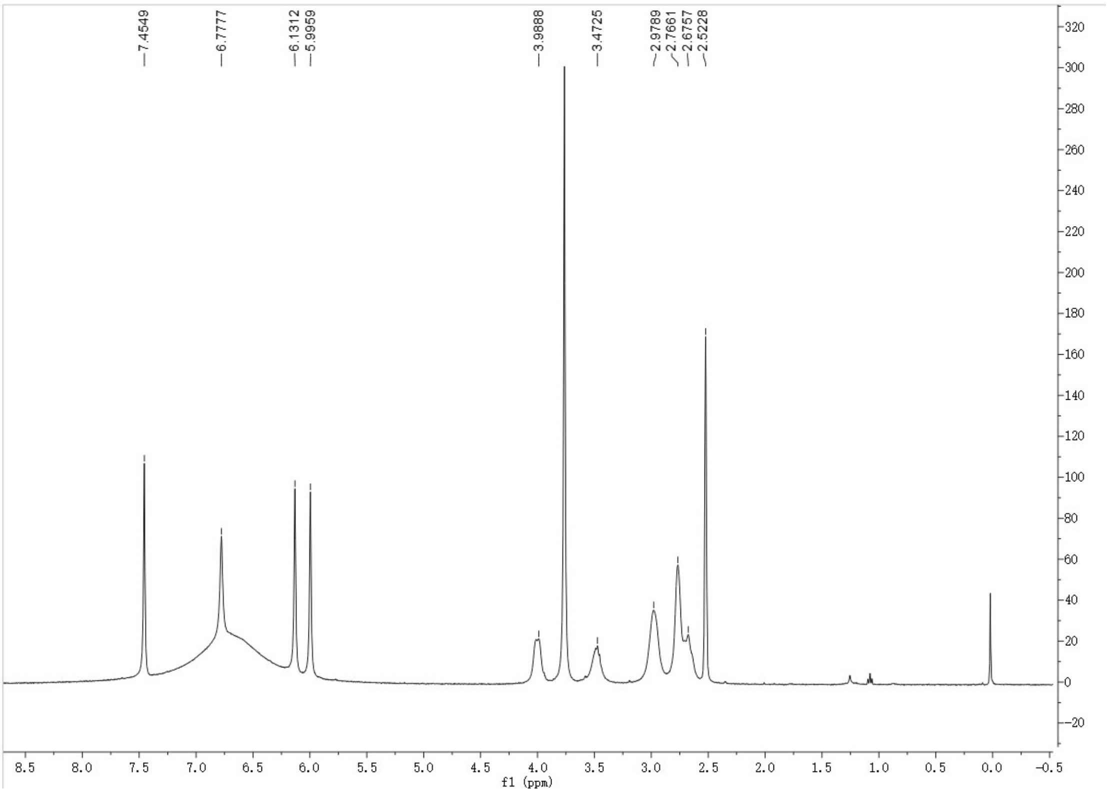

Figure S15. ^{1}H NMR Spectrum of 3-Demethylcassythine

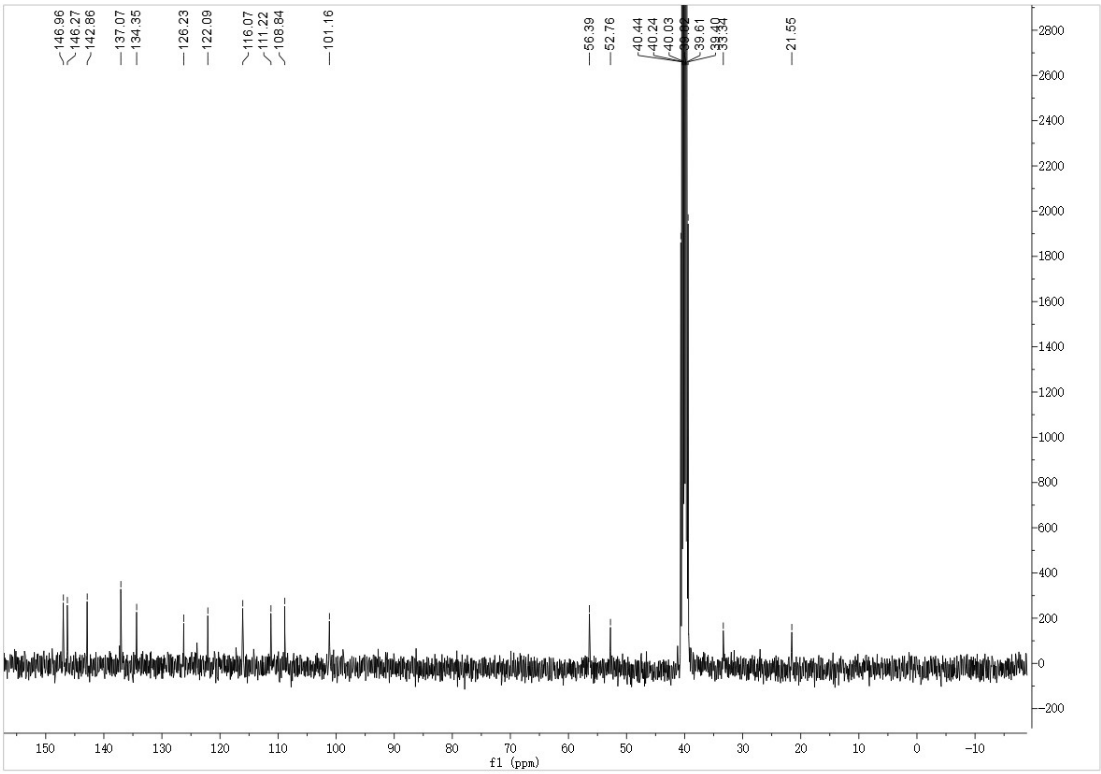

Figure S16. ^{13}C NMR Spectrum of 3-Demethylcassythine

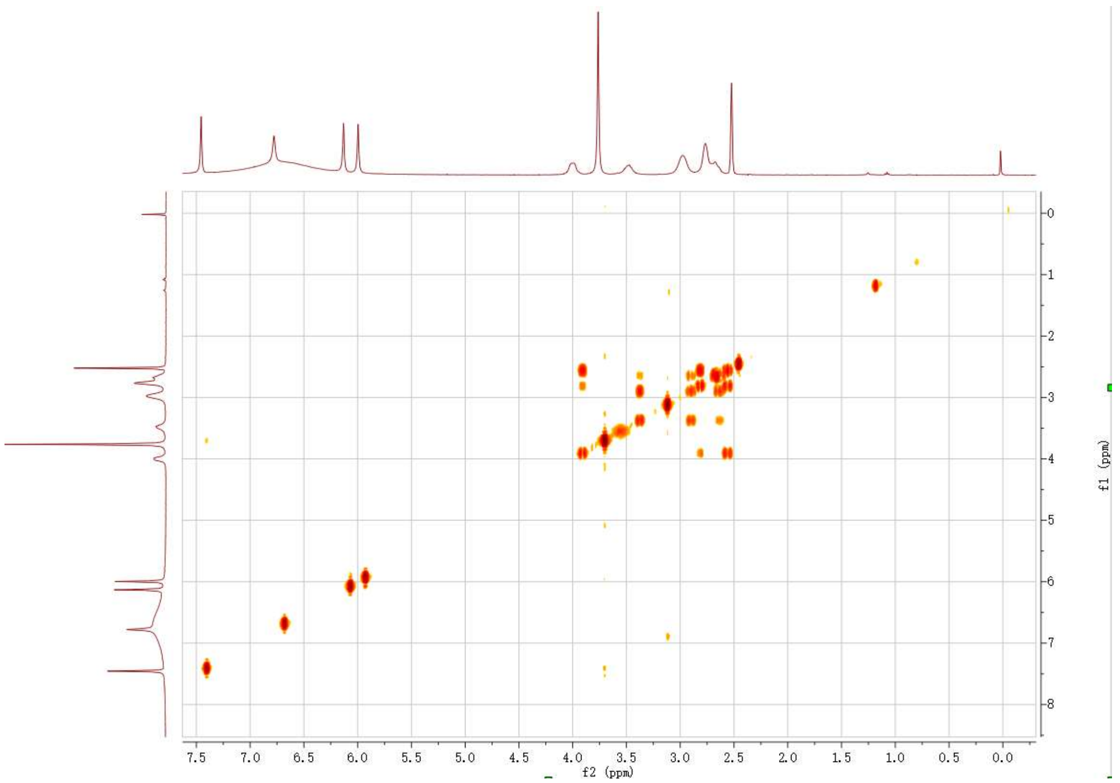

Figure S17. ^{1}H-^{1}H COSY Spectrum of 3-Demethylcassythine

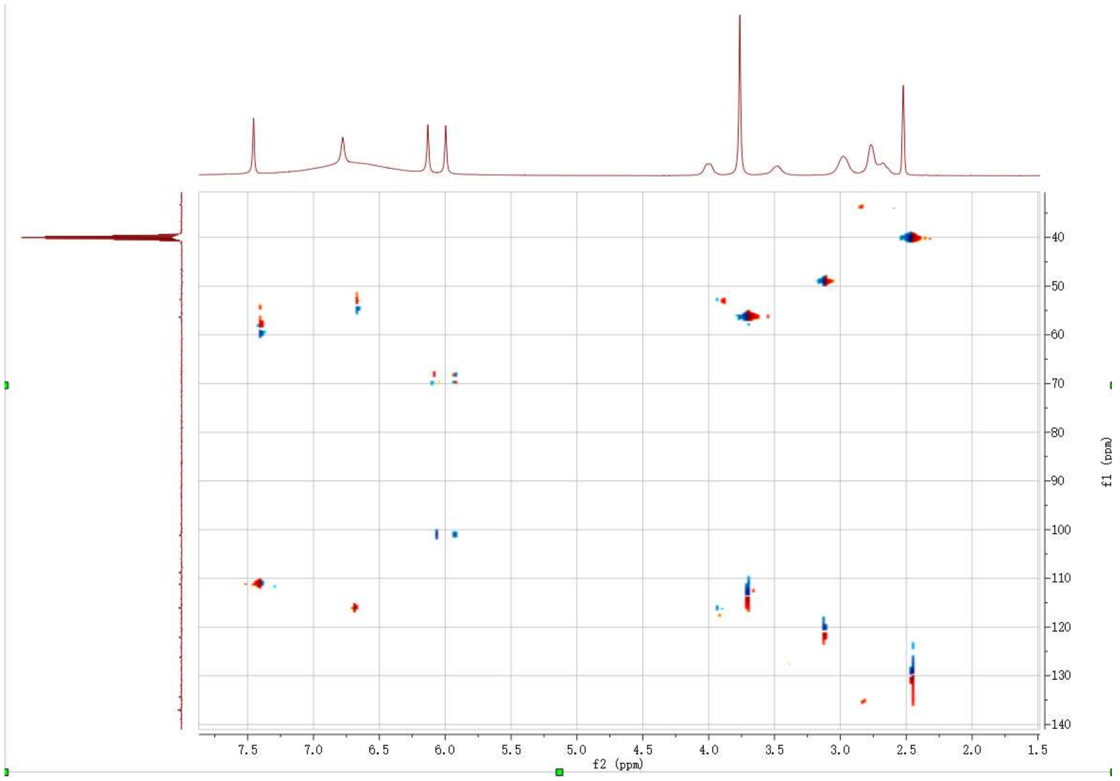

Figure S18. HSQC Spectrum of 3-Demethylcassythine

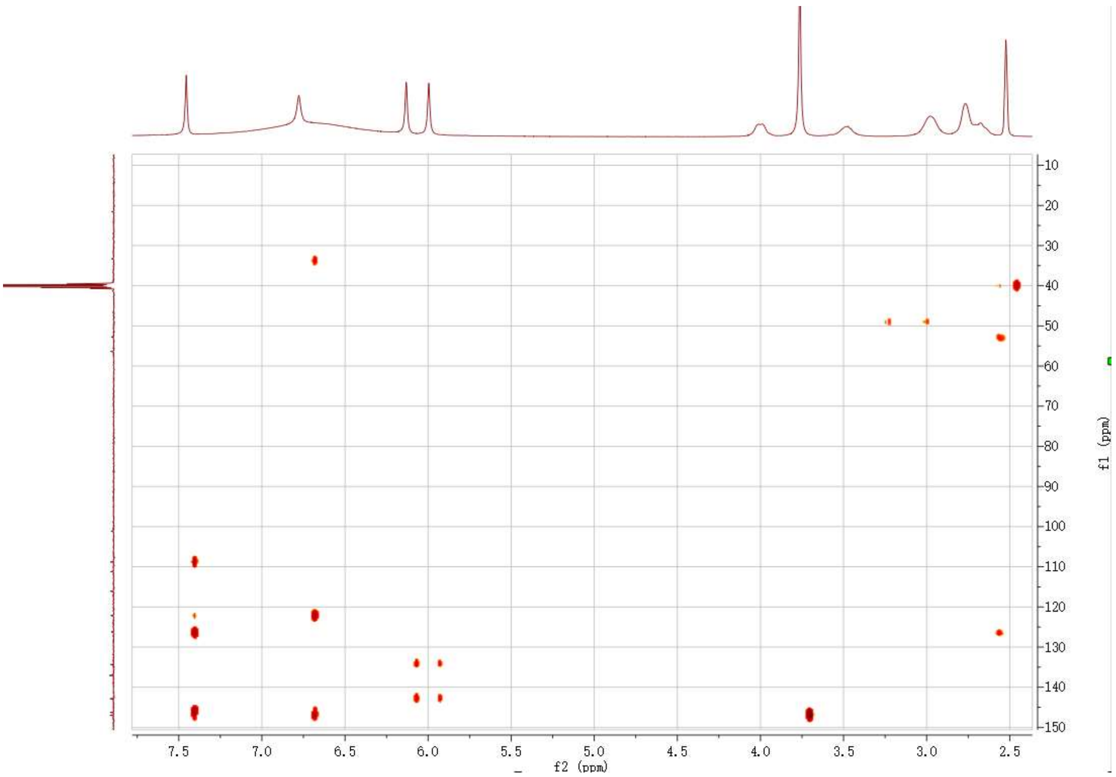

Figure S19. HMBC Spectrum of 3-Demethylcassythine

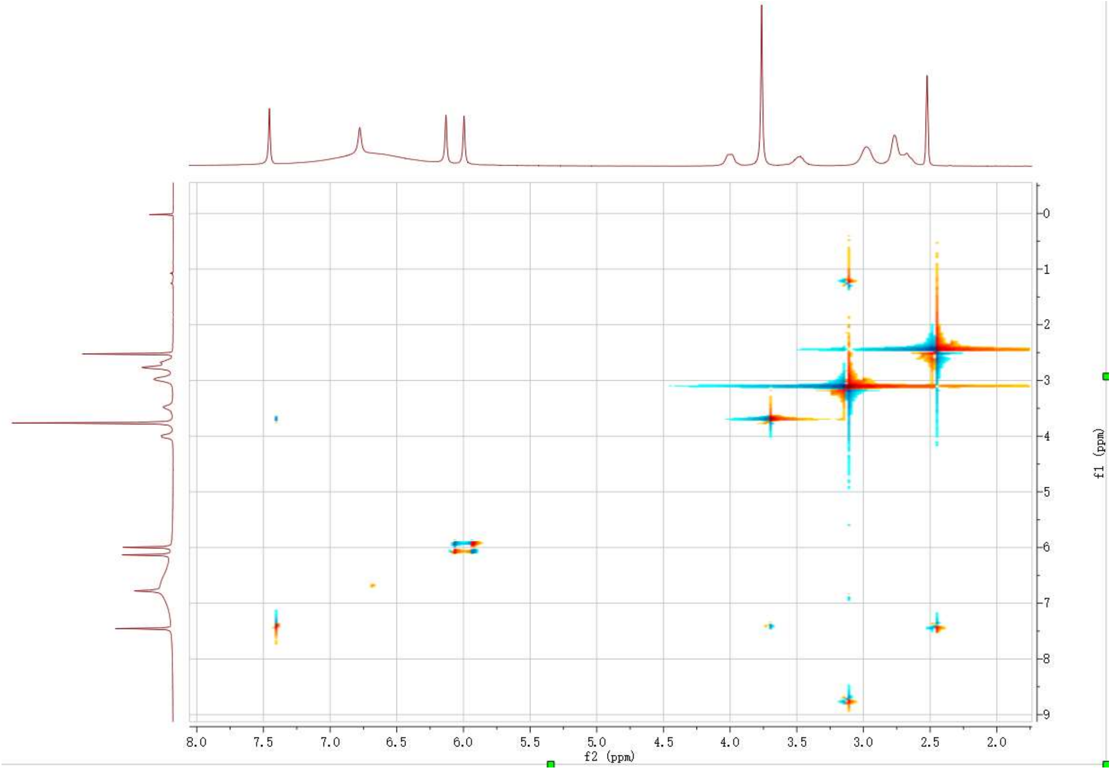

Figure S20. NOESY Spectrum of 3-Demethylcassythine

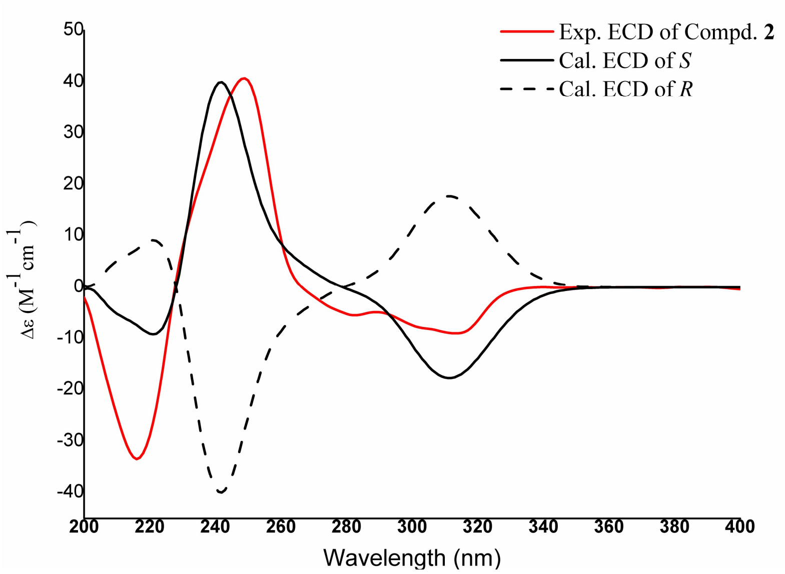

Figure S21. Experimental and Calculated ECD Spectra of 3-Demethylcassythine

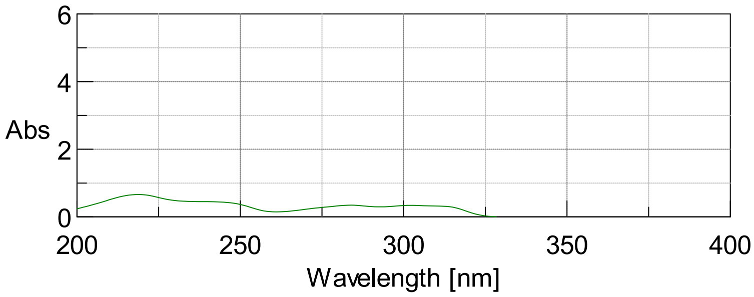

Figure S22. UV-Vis Absorption Spectrum of *N*-demethyl

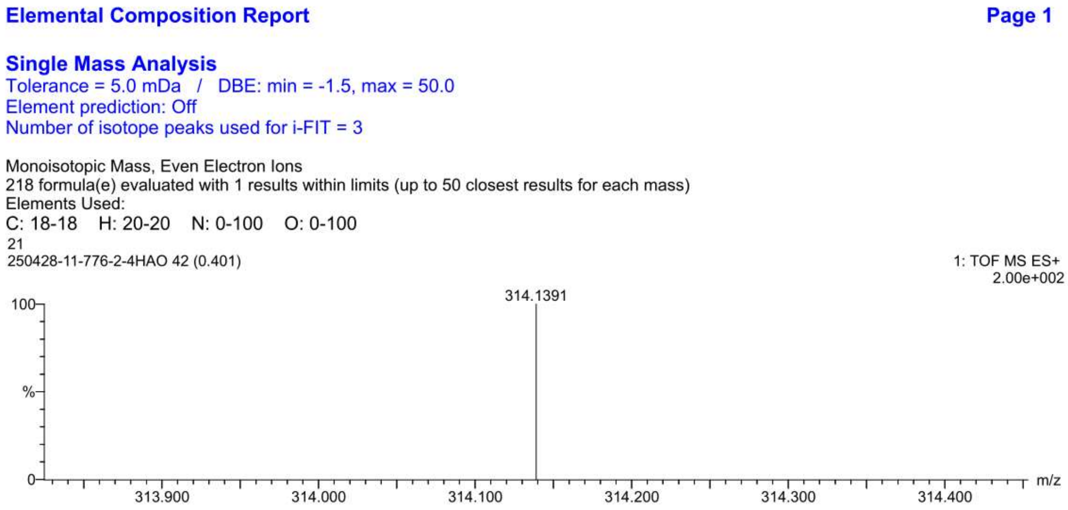

Figure S23. Mass Spectrum of *N*-demethyl

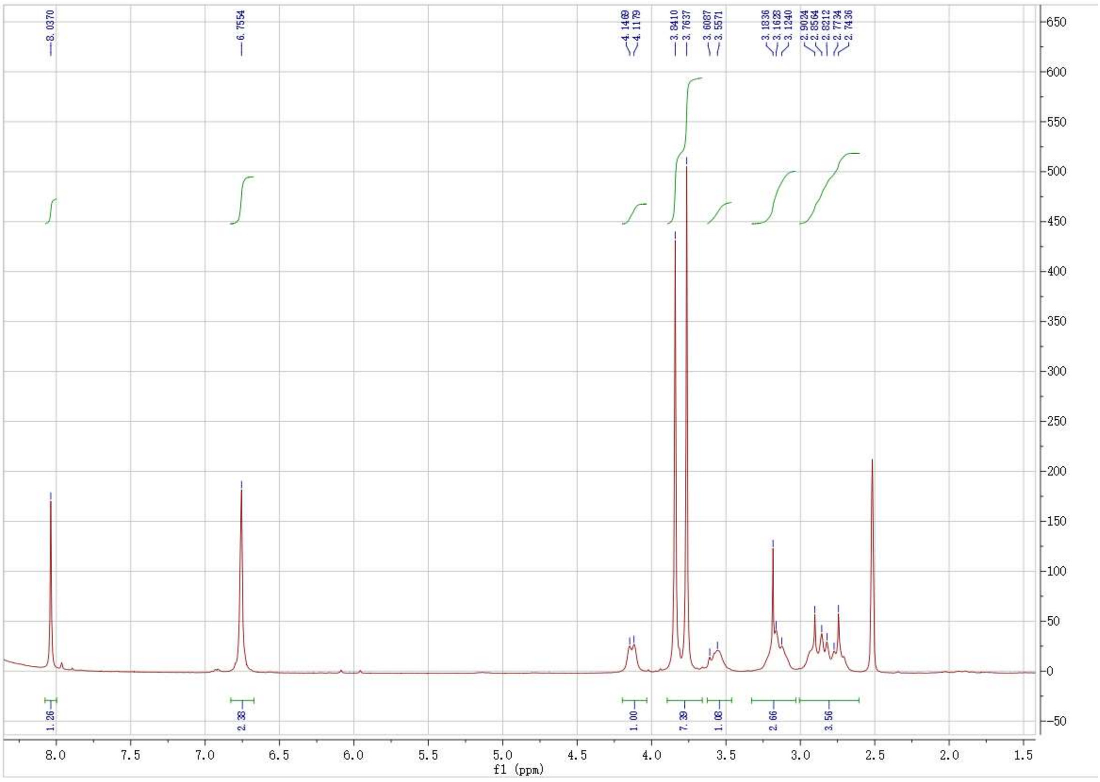

Figure S24. ^{1}H NMR Spectrum of *N*-demethyl

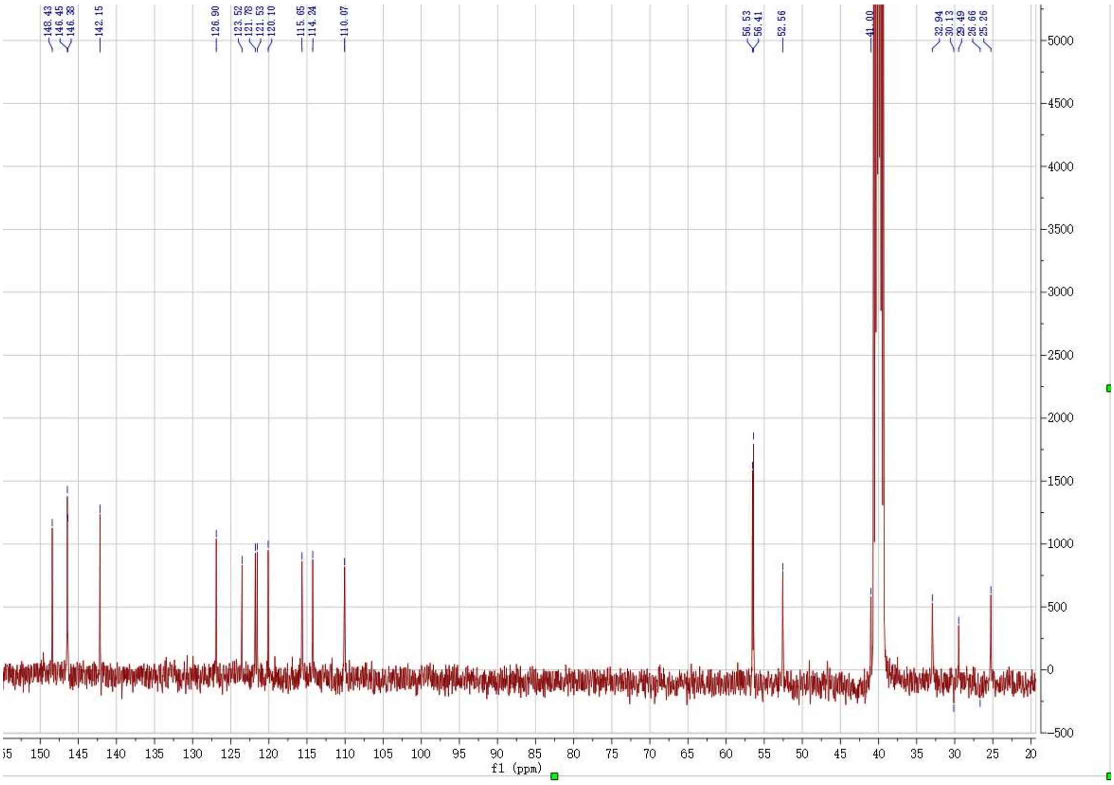

Figure S25. ^{13}C NMR Spectrum of *N*-demethyl

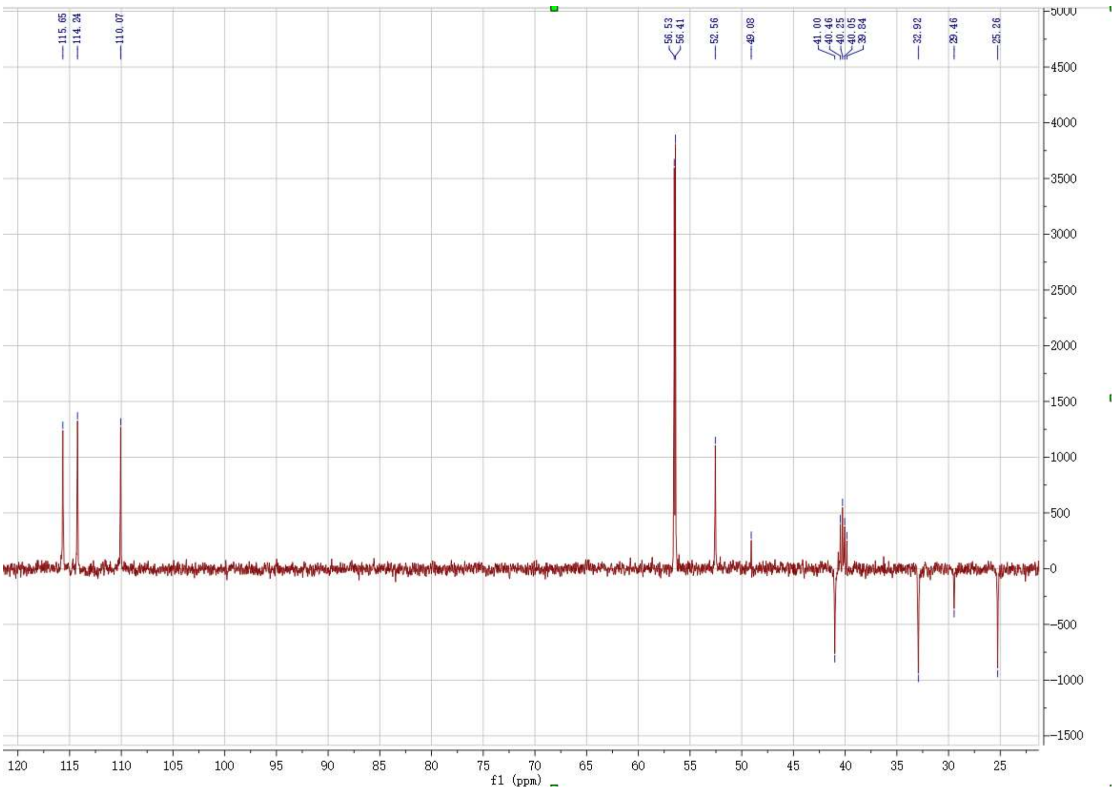

Figure S26. DEPT Spectrum of *N*-demethyl

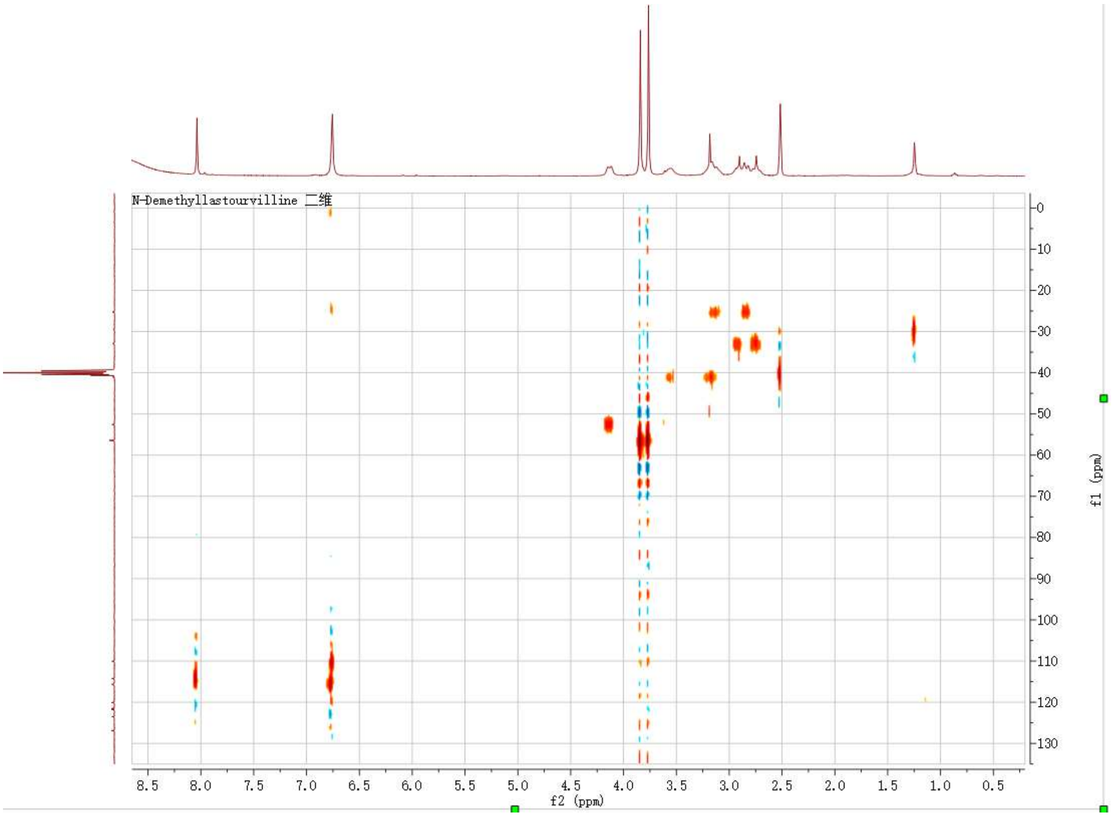

Figure S27. HSQC Spectrum of *N*-demethyl

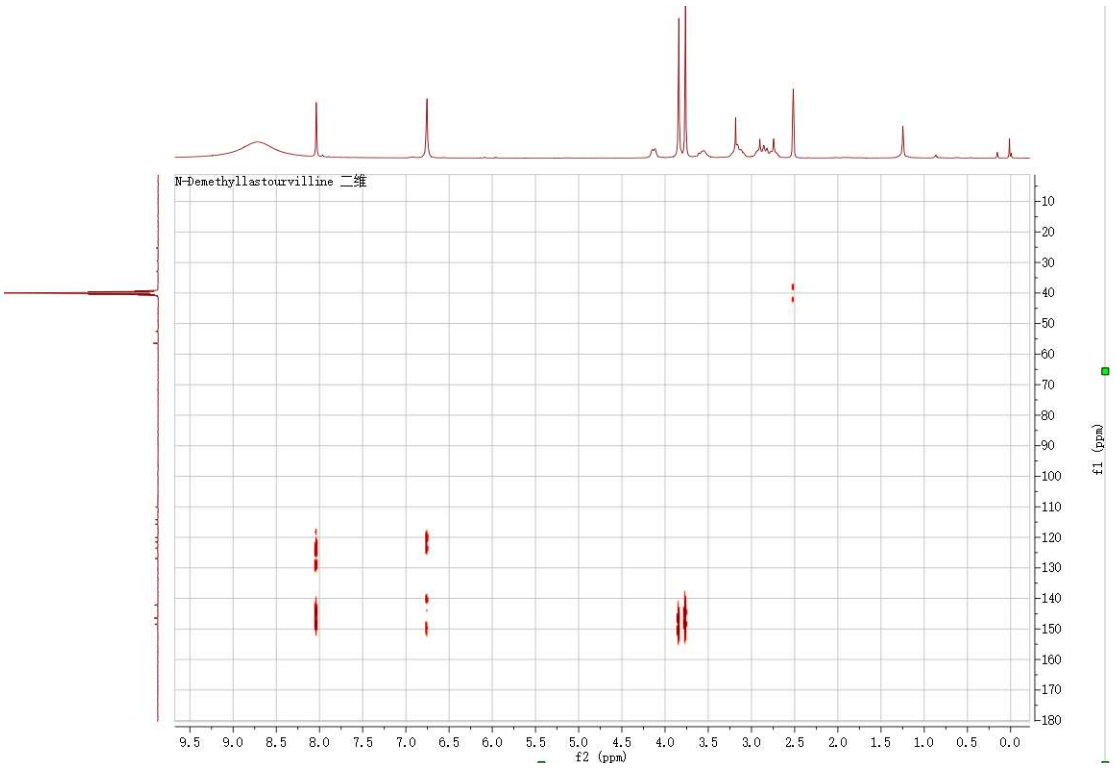

Figure S28. HMBC Spectrum of *N*-demethyl

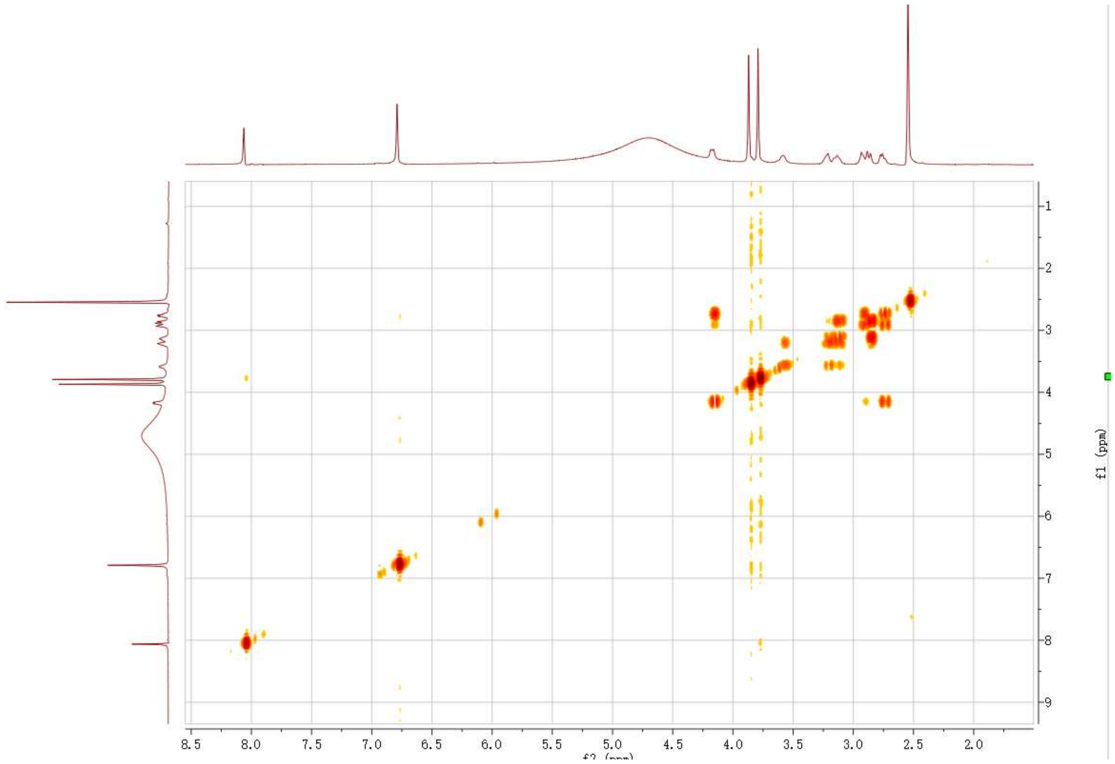

Figure S29. ^{1}H-^{1}H COSY Spectrum of *N*-demethyl

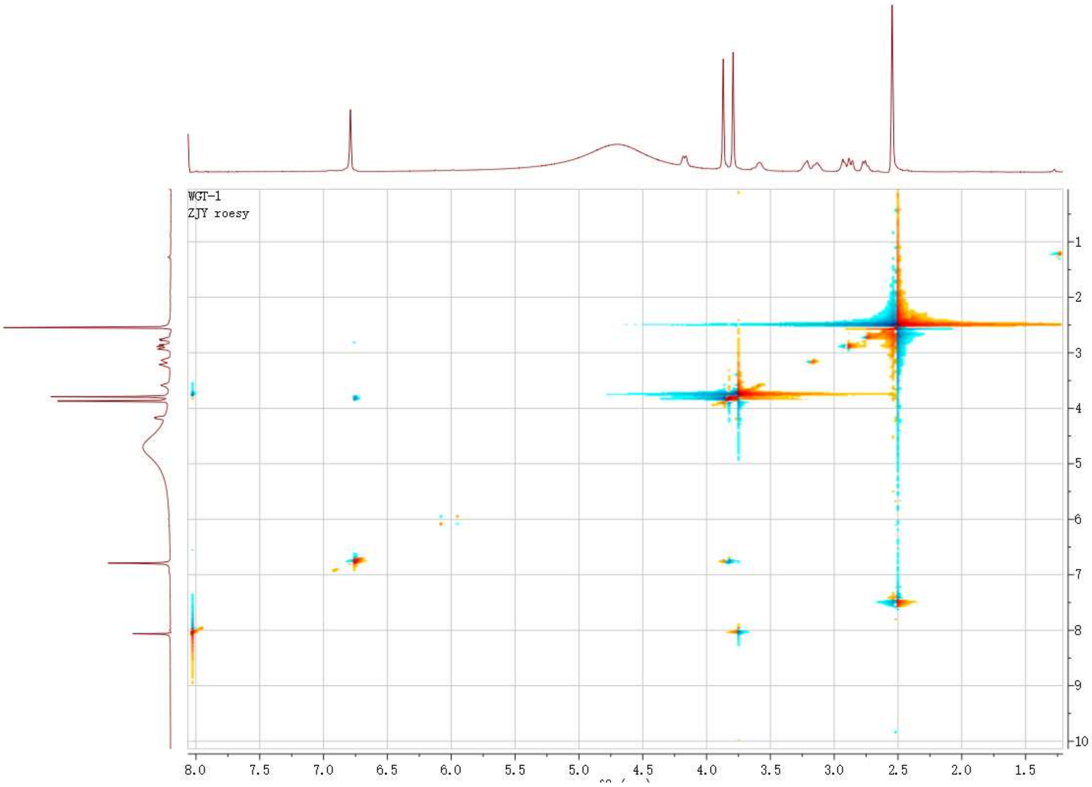

Figure S30. NOESY Spectrum of *N*-demethyl

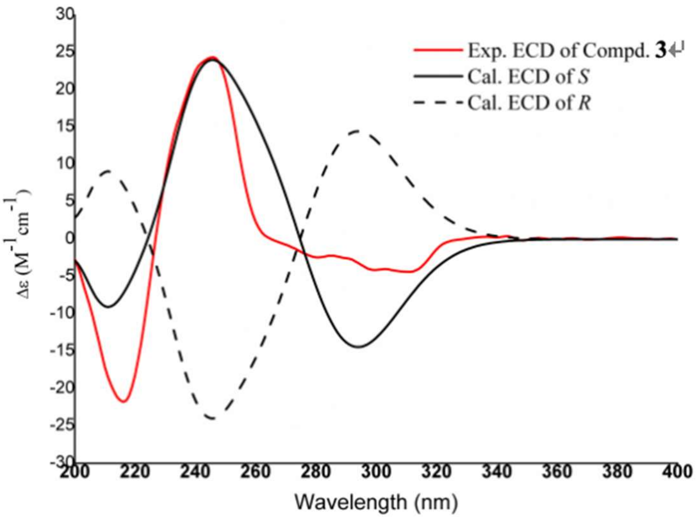

Figure S31. Experimental and Calculated ECD Spectra of *N*-demethyl
